# Trajectories of State-Level Sepsis-Related Mortality by Race and Ethnicity Group in the United States

**DOI:** 10.3390/jcm13102848

**Published:** 2024-05-12

**Authors:** Lavi Oud, John Garza

**Affiliations:** 1 Division of Pulmonary and Critical Care Medicine, Department of Internal Medicine, Texas Tech University Health Sciences Center at the Permian Basin, 701 W. 5th Street, Odessa, TX 79763, USA; 2 Texas Tech University Health Sciences Center at the Permian Basin, 701 W. 5th Street, Odessa, TX 79763, USA; garza_j@utpb.edu; 3 Department of Mathematics, The University of Texas of the Permian Basin, 4901 E. University Blvd, Odessa, TX 79762, USA

**Keywords:** disparity, ethnicity, mortality, race, sepsis, states

## Abstract

**Background:** Recent reports on the national temporal trends of sepsis-related mortality in the United States (US) suggested improvement of outcomes in several race and ethnicity groups. However, it is unknown whether national data reflect state-level trajectories. **Methods:** We used the Centers for Disease Control and Prevention Wide-ranging Online Data for Epidemiologic Research Multiple Cause of Death data set to identify all decedents with sepsis in the US during 2010–2019. Negative binomial regression models were fit to estimate national and state-level trends of age-adjusted sepsis-related mortality rates within race and ethnicity groups. **Results:** There were 1,852,610 sepsis-related deaths in the US during 2010–2019. Nationally, sepsis-related mortality rates decreased among Blacks and Asians, were unchanged among Hispanics and Native Americans, and rose among Whites. The percent of states with similar trends were 30.0% among Blacks, 32.1% among Asians, 74.3% among Hispanics, 75.0% among Native Americans, and 66.7%% among Whites, while trending in opposite direction from 3.6% among Asians to 15.0% among Blacks. **Conclusions:** National trends in sepsis-related mortality in the US did not represent state-level trajectories in race ethnicity groups. Gains in sepsis outcomes among race and ethnicity groups at the national level were not shared equitably at the state level.

## 1. Introduction

Sepsis remains a leading cause of mortality, estimated to contribute to nearly 1 in 5 deaths worldwide in 2017 [1]. However, the death toll of sepsis varies across populations. In the United States (US), disparities in the sepsis-related death toll across race and ethnicity groups, affecting predominantly racial and ethnic minorities, have been extensively documented [2,3]. However, evidence has been scarce on the temporal trends of sepsis-related mortality within race and ethnicity groups in the US. A recent study by Prest and colleagues showed, encouragingly, that sepsis-related mortality decreased among several race and ethnicity groups, though rising in others [4]. These data are important for gauging the evolution of the epidemiology of sepsis and the impact of national efforts to reduce its burden. However, national estimates of sepsis-related mortality in the US do not represent state-level data, with the latter shown in cross-sectional studies to vary substantially across states within some racial groups [5]. Data are lacking, however, on how sepsis-related mortality has changed over time within and across race and ethnicity groups in the US when examined at the state level, and whether national-level trends reflect state-level trajectories.

Sepsis-related mortality is a function of both the risk of sepsis (which drives its incidence) and case fatality among septic patients. Thus, for instance, sepsis-related mortality of a population can remain unchanged over time, while sepsis incidence rises but sepsis-related case fatality decreases. Factors reported to be associated with the risk of sepsis include both personal (e.g., demographic [6], genetic [7], health behaviors [8], and comorbid conditions [9]) and community-level (e.g., social vulnerability [10], access to health care [11], urbanicity [12], and environmental exposures [13]) domains. The factors associated with case fatality among septic patients generally include similar personal [3,8,14] and community-level [11,15,16,17] domains as those associated with the risk of sepsis, as well as infection characteristics [18,19], severity of illness [18], processes of care [20], and hospital characteristics [21,22,23,24]. Variations in exposure to these factors across race and ethnicity groups may contribute to the documented race and ethnicity-related disparities in sepsis-related mortality [25,26]. However, the contemporary national regulatory [27] and professional guideline-based [28] efforts to reduce the burden of sepsis have been largely focused on processes of care of patients with sepsis. Nevertheless, beyond the critical role of optimized processes of care in septic patients, many of the aforementioned factors reported to be associated with the risk of sepsis and its case fatality are also potentially modifiable. Indeed, sepsis is increasingly considered to represent a public health problem that requires a population- and system-based approach [29].

Better understanding of the state-level dynamics of sepsis-related mortality within race and ethnicity groups may refine the epidemiological perspectives provided by the national trends and can inform more targeted efforts to reduce disparities in the death toll. In this study of the national repository of mortality data in the US, our primary objective was to characterize the temporal trends of state-level sepsis-related mortality within race and ethnicity groups against the corresponding national trajectories. Our secondary objectives were to examine changes in disparities in sepsis-related mortality across states within race and ethnicity groups and in disparities in the gaps of within-state sepsis-related mortality between racial and ethnic minority groups and White individuals across states.

## 2. Materials and Methods

This was a retrospective, population-based study. The study was determined to be exempt from formal review by the Texas Tech Health Sciences Center’s Institutional Review Board because we used a publicly available, de-identified data set, and thus was not considered human research in accordance with 45 CFR 46.101(c). This study followed the STROBE guidelines on reporting observational studies in epidemiology [30].

### 2.1. Data Sources and Study Population

Death certificate data were obtained from the multiple cause-of-death (MCOD) records of the National Center of Health Statistics (NCHS), accessed through the Centers for Disease Control and Prevention Wide-ranging Online Data for Epidemiological Research (CDC WONDER) database [31]. CDC WONDER mortality data, based on information from all death certificates filed in the 50 states and the District of Columbia, includes demographic data and up to 20 causes of death, reported in aggregate. Causes of death are reported using the *International Classification of Diseases*, *Tenth Revision*, *Clinical Modification* (ICD-10) codes. The information in death certificates, excluding race and ethnicity, is completed by the certifier (e.g., physician, medical examiner, or coroner) and is then coded by the NCHS. The race and ethnicity information documented in the death certificate relies primarily on the input of the funeral director, a report by an informant, or, in the absence of an informant, physical observation. The race and ethnicity data of decedents collected from death certificates were reported in accordance with statutes set forth by the US Office of Management and Budget of the federal government [32], and were categorized into 5 mutually exclusive categories: non-Hispanic Black or African American (hereafter, Black), non-Hispanic Asian or Pacific Islander (hereafter, Asian), non-Hispanic American Indian or Alaska Native (hereafter, Native American), non-Hispanic White (hereafter, White), and Hispanic or Latino (hereafter, Hispanic). Population data were provided via CDC WONDER, based on the US Bureau of the Census estimates of the total US and state resident populations for each race and ethnicity group. The year 2010 population is based on a census count on April 1. The years 2011–2019 population data are based on postcensal estimates of the July 1 resident population of each year.

We identified all decedents with a diagnosis of sepsis in any position in the MCOD data during 2010–2019, using ICD-10 codes A02.1, A20.7, A22.7, A26.7, A32.7, A40.0-A40.9, A41.0-A41.9, A42.7, and B37.7, similar to prior studies [4,33]. The CDC WONDER data set did not include ICD-10 codes for severe sepsis (R65.20) or septic shock (R65.21).

Age-adjusted mortality rates were provided by CDC WONDER, are age-standardized to the 2000 US standard population using the direct method and presented per 100,000 population. The NCHS considers death rates based on counts of less than 20 as statistically unreliable, and data output of mortality rates below this count threshold are flagged as “Unreliable” in CDC WONDER with no numeric estimate. In addition, subnational statistics reporting fewer than 10 persons were suppressed by the NCHS due to confidentiality constraints, in order to protect personal privacy. These restrictions of mortality rate data in the CDC WONDER database affected racial and minority groups in this study due to their often small populations in specific states, with similar constraints reported in prior epidemiological studies of sepsis [5] and other conditions [34] using this data set.

### 2.2. Statistical Analysis

We summarized categorical variables as frequencies and percentages, while continuous variables were reported as median (interquartile range [IQR]). The Mann–Whitney test was used for comparison of continuous variables.

We modeled the temporal trends of sepsis-related mortality rates using negative binomial regression with log-link and robust standard errors, and with population offset. Changes in sepsis-related mortality rates are expressed as annual percent change (APC) and 95% confidence intervals (95% CI), representing the summary measure from 2010 to 2019 overall and stratified by race and ethnicity. Choropleth maps were used to illustrate state-level trends of sepsis-related mortality overall and within race and ethnicity groups.

We have examined two measures of disparity in sepsis-related mortality across states: (a) inequalities in sepsis-related mortality rates *within* individual race and ethnicity groups and (b) inequalities in the gap in sepsis-related mortality rates *between* individual racial and ethnic minority groups and White individuals in a given state. The span of inequality in cross-state sepsis-related mortality within individual race and ethnicity groups was quantified as the difference in mortality rates between states with the highest vs. lowest values in each group. The span of inequality in the across-state gap in within-state sepsis-related mortality between individual racial and ethnic minority groups and those of White individuals was quantified as the difference in the values of this gap between states with the highest vs. lowest values for each racial and ethnic minority–White individuals’ dyad. Changes in the summary estimates of both inequality measures across states over the study period were examined, comparing values for the years 2010 and 2019.

Because the introduction of the third international consensus definition of sepsis (Sepsis-3) in early 2016 [35] may have affected clinicians’ diagnosis of sepsis starting that year, which could have affected trends in sepsis mortality spanning 2016, we performed sensitivity analyses using interrupted time series analyses (ITS), fitting segmented negative binomial regression models, comparing the periods of 2010–2015 vs. 2016–2019, and, separately, the periods of 2010–2014 vs. 2015–2019 for the national-level overall US population. We have used this combined approach to examine whether any trend changes in sepsis-related mortality rates observed across the year 2016 can be potentially ascribed to that year’s introduction of the Sepsis-3 definitions, rather than being incidental. Specifically, we first we examined a baseline time trend (the years 2010–2015), level (intercept) change (in 2016), time trend during 2016–2019 (that is, once Sepsis-3 definitions were introduced), and change of time trends between 2010–2015 and 2016–2019. We then repeated the model to examine a baseline time trend (the years 2010–2014), level (intercept) change (in 2015), time trend during 2015–2019, and change of time trends between 2010–2014 and 2015–2019. Similarly to the primary models, trends in sepsis-related mortality are expressed as annual percent change and 95% CI of sepsis-related mortality rates, while the coefficients for level changes and changes between trends are expressed as percentages and 95% CIs.

Data management was performed using Microsoft Excel 2010 (Microsoft, Redmond, Washington) and statistical analyses were performed with R 4.0.5 (R Foundation for Statistical Computing, Vienna, Austria). A 2-sided *p* value < 0.05 was considered statistically significant.

## 3. Results

A total of 1,852,610 sepsis-related deaths among US residents were reported during 2010–2019, of which 272,218 (14.7%) were Black individuals, 154,415 (8.3%) were Hispanic individuals, 52,019 (2.8%) were Asian individuals, 15,267 (0.8%) were Native American individuals, and 1,352,873 (73.0%) were White individuals. Of the 50 US states (and the District of Columbia), our sample included 40 states (and the District of Columbia) for Black individuals, 35 states for Hispanic individuals, 28 states for Asian individuals, 16 states for Native American individuals, and 50 states (and the District of Columbia) for White individuals. These states represented 99.4% of the Black population, 97.8% of the Hispanic population, 94.7% of the Asian population, 74.9% of the Native American population, and 100% of the White population.

The national-level temporal trends in sepsis related mortality rates for the US population and for individual race and ethnicity groups are presented in Table 1. Sepsis-related mortality rose over time for the whole US population, decreased among Black and Asian individuals, remained unchanged among Hispanic and Native American individuals, and rose among White individuals.

### 3.1. Trends of State-Level Sepsis-Related Mortality

**Figure 1:**
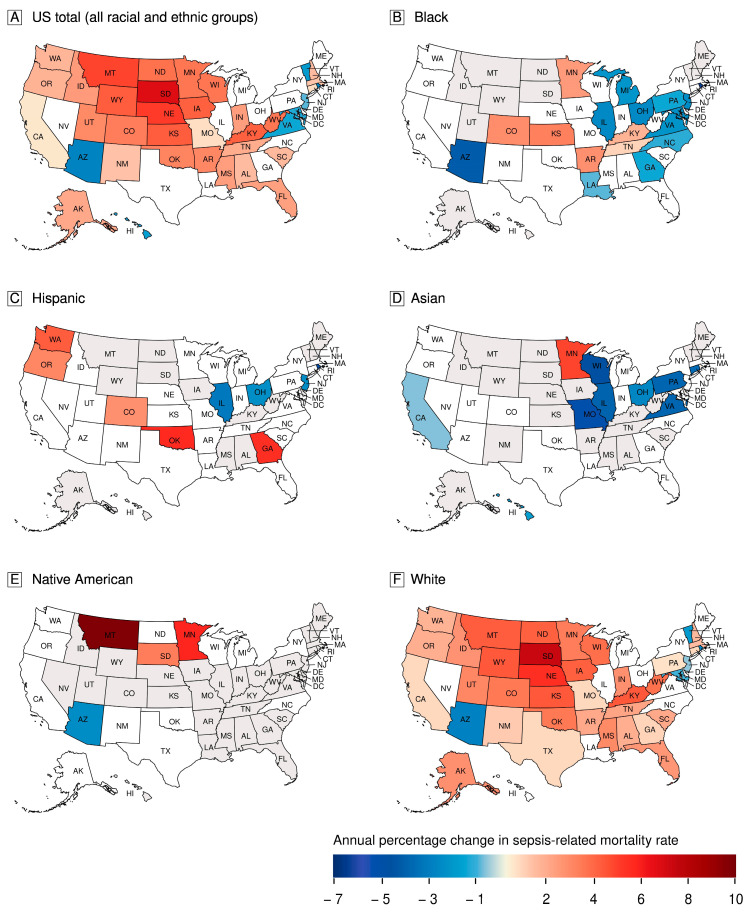
Temporal trends of state-level sepsis-related mortality in the United States, 2010–2019. The maps show the direction and rate of change in age-adjusted sepsis-related mortality rates in individual states, expressed as annual percent change for the United States population (**A**) and for 5 mutually exclusive race and ethnicity groups (**B**–**F**). Red colors represent states with rising sepsis-related mortality rates and blue colors represent states with decreasing sepsis-related mortality rates during 2010–2019. White color represents states with no statistically significant changes in the annual sepsis-related mortality rates. Gray color represents states where reliable age-adjusted sepsis-related mortality rates could not be determined.

State-level trends of sepsis-related mortality rates for the US population and race and ethnicity groups are presented in Figure 1 and Tables S1–S6.

For the US population, sepsis-related mortality rates increased in 31 (61.0%) states, decreased in 8 (16.0%) states (and the District of Columbia), and were unchanged statistically in 12 (23.0%) states. Among states with mortality rate data, the direction of sepsis-related mortality rates during 2010–2019 varied within each race and ethnicity group. Among Black individuals, sepsis-related mortality rates increased in 6 (15%) states, decreased in 12 (30.0%) states, and were unchanged in 22 (55.0%) states (and the District of Columbia). Among Hispanic individuals, sepsis-related mortality rates increased in 5 (14.3%) states, decreased in 4 (11.4%) states, and were unchanged in 26 (74.3%) states.

Among Asian individuals, sepsis-related mortality rates increased in 1 (3.6%) state, decreased in 9 (32.1%) states, and were unchanged in 18 (64.3%) states. Among Native American individuals, sepsis-related mortality rates increased in 3 (18.8%) states, decreased in 1 (6.2%) state, and were unchanged in 12 (75.0%) states. Among White individuals, sepsis-related mortality rates increased in 34 (66.7%) states, decreased in 6 (11.7%) states (and the District of Columbia), and were unchanged in 11 (21.6%) states.

The annual rate of change in state-level sepsis-related mortality rates varied widely within all race and ethnicity groups, being as high as APC 9.8% (95% CI 6.2% to 13.5%; *p* < 0.0001) among Native American individuals in Montana in states with rising mortality rates, and as low as APC −6.4% (95% CI −10.3% to −2.3%; *p* = 0.0023) among Black individuals in Rhode Island in states with decreasing mortality rates.

### 3.2. Inequalities in Sepsis-Related Mortality across States

**Figure 2:**
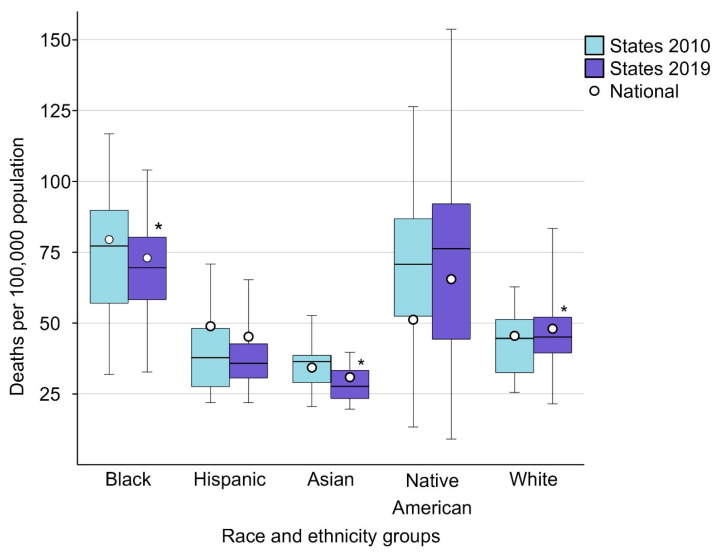
Changes in cross-state differences in sepsis-related mortality from 2010 to 2019 within individual race and ethnicity groups. The bottom border, middle line, and top border of the boxes indicate the 25th, 50th, and 75th percentiles, respectively, of age-adjusted sepsis-related mortality rates across all states with available mortality rate data for each race and ethnicity group; whiskers represent the full range of sepsis-related mortality rates across states, and circles represent the national-level sepsis-related mortality rates for each race and ethnicity group. Box and whiskers, as well as the circle data are presented for each race and ethnicity group for the years 2010 and 2019. Asterisks denote race and ethnicity groups with statistically significant change in cross-state sepsis-related mortality rates from 2010 to 2019.

Data on the inequalities in sepsis-related mortality rates across states within individual race and ethnicity groups and their change from 2010 to 2019 are presented in Figure 2 and in Table S7. Data on the change in cross-state sepsis-related mortality rates for each state within race and ethnicity groups are detailed in Tables S2–S6.

The difference in sepsis-related mortality rates (per 100,000 population) across states within individual race and ethnicity groups ranged from 32.2 among Asian individuals to 113.1 among Native American individuals in 2010, and from 20.1 among Asian individuals to 144.6 among Native American individuals in 2019.

The median value of sepsis-related mortality rates (per 100,000 population) across states within individual race and ethnicity groups decreased between 2010 and 2019 from 77.8 to 69.6 (*p* = 0.0032) among Black individuals; from 36.5 to 27.7 (*p* = 0.0004) among Asian individuals; and increased from 44.6 to 45.1 (*p* = 0.0031) among White individuals. Across-state sepsis-related mortality rates did not change significantly among Hispanic individuals (*p* = 0.1518) and Native American individuals (*p* = 0.8564).

By 2019, across-state sepsis-related mortality rates (per 100,000 population) varied widely within each racial and ethnic group, ranging among Black individuals from 32.7 (Arizona) to 104.0 (District of Columbia), from 21.9 (Missouri) to 65.3 (Texas) among Hispanic individuals, from 19.6 (Arizona) to 39.7 (Texas) among Asian individuals, from 9.1 (Texas) to 153.7 (South Dakota) among Native American individuals, and from 21.5 (Hawaii) to 83.4 (Kentucky) among White individuals.

### 3.3. Inequalities in Within-State Sepsis-Related Mortality Gaps across States

**Figure 3:**
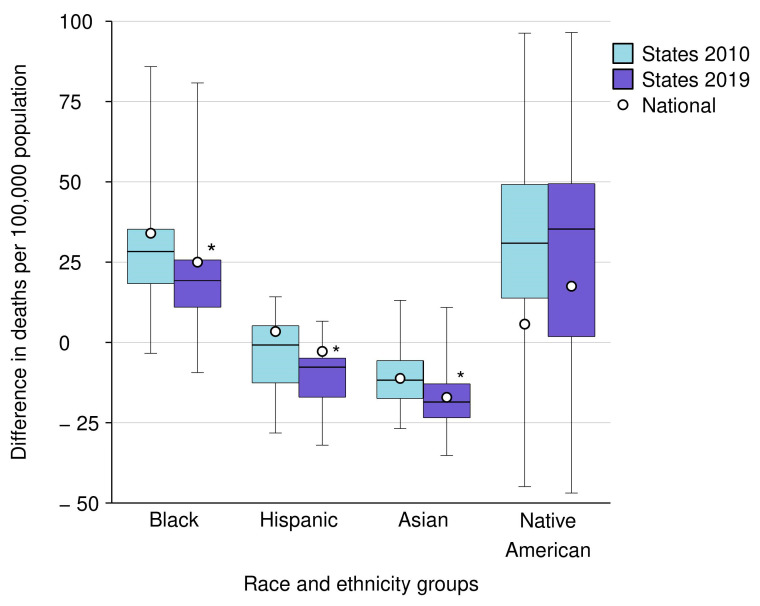
Changes in cross-state differences in the gap of within-state sepsis-related mortality between individual race and ethnicity minority groups and White individuals, from 2010 to 2019. The bottom border, middle line, and top border of the boxes indicate the 25th, 50th, and 75th percentiles, respectively, of the difference in age-adjusted sepsis-related mortality rates between individual race and ethnicity minority groups and White individuals within all states with available mortality rate data for both; whiskers represent the full range of the difference in sepsis-related mortality rates within states, and circles represent the national-level differences in sepsis-related mortality rates between individual race and ethnicity minority groups and White individuals. Box and whiskers, as well as the circle data, are presented for each racial and ethnic minority group for the years 2010 and 2019. Asterisks denote race and ethnicity groups with statistically significant changes in the within-state differences in sepsis-related mortality rates between individual race and ethnicity minority groups and White individuals from 2010 to 2019.

The data on inequalities in within-state sepsis-related mortality gaps between racial and ethnic minority groups and White individuals across states and their change from 2010 to 2019 are presented in Figure 3 and Table S8, and are detailed at the state level in Tables S9–S12.

The difference in the gaps of within-state sepsis-related mortality rates (per 100,000 population) between racial and ethnic minority groups and White individuals across states ranged from 39.9 among Asian individuals to 141.2 among Native American individuals in 2010, and from 38.6 among Hispanic individuals to 146.1 among Native American individuals in 2019.

The median value of the gaps of within-state sepsis-related mortality rates (per 100,000 population) between racial and ethnic minority groups and White individuals across states decreased between 2010 and 2019 among Black individuals from +28.3 to +19.3 (*p* < 0.0001); from −0.8 to −7.7 (*p* = 0.0003) among Hispanic individuals; and from −11.8 to −18.6 (*p* = 0.0023) among Asian individuals, but did not change statistically among Native American individuals (+30.9 to +35.3; *p* = 0.4037).

By 2019, the gaps in within-state sepsis-related mortality rates (per 100,000 population) between racial and ethnic minority groups and White individuals varied widely across states, ranging among Black individuals from −9.4 (Rhode Island) to +80.8 (District of Columbia), from −32.2 (Arkansas) to +6.6 (Texas) among Hispanic individuals, from −35.2 (Oklahoma) to +10.9 (Hawaii) among Asian individuals, and from −49.6 (Texas) to +96.5 (South Dakota) among Native American individuals.

Among states with available sepsis-related mortality rate data for both racial and ethnic minority groups and White individuals, by 2019 within-state sepsis-related mortality rates were higher among Black individuals in 37 (93.0%) states (and the District of Columbia) and lower in 3 (7.0%) states (Massachusetts, New Mexico, and Rhode Island); higher in 5 (14%) states and lower in 30 (86%) states among Hispanic individuals; higher in 2 (7.0%) states and lower in 26 (93%) states among Asians; and higher in 13 (81%) states and lower in 3 (19%) states (California, New York, and Oregon) among Native American individuals.

Sensitivity analyses showed sepsis-related mortality rose gradually over time for the whole US population and subsequently plateaued mid-decade. The detailed data of ITS analyses are presented in Tables S13 and S14. The ITS analysis of the national-level US population, comparing the time trends of sepsis-related mortality between 2010–2015 and 2016–2019, showed that the mortality trend was rising (*p* < 0.0001) and then plateaued (*p* = 0.9820), respectively, with a significant change in trends (coefficient −4.5 [95% CI −6.6 to −2.3]; *p* = 0.0001). There was no discontinuity (no significant level change) in sepsis-related mortality rates between the years 2015 and 2016 (coefficient 4.3 [95% CI −0.9 to 9.8]; *p* = 0.1081). On repeating the ITS analysis, comparing the time trends of sepsis-related mortality for the national-level US population between 2010–2014 and 2015–2019, findings were similar, with the mortality trend rising (*p* < 0.0001) and then plateauing (*p* = 0.9916), respectively, again with a significant change in trends between the two periods (coefficient −3.3 [95% CI −4.9 to −1.6]; *p* = 0.0001).

## 4. Discussion

In this analysis of national data from death certificates in the US during the last decade, the direction and rate of change in state-level sepsis-related mortality varied considerably within and across all examined race and ethnicity groups. In addition, although overall inequalities in both across-state sepsis-related mortality within individual race and ethnicity groups and in the gaps of within-state differences in sepsis-related mortality between racial and ethnic minorities and White individuals decreased in several groups by the end of the last decade, substantial disparities remained within each group. To the best of our knowledge, this analysis is the first to provide state-level estimates of the trajectories of sepsis-related mortality and changes in cross-state disparities in sepsis-related death toll in these populations, and lack of previous studies in this area in the US precluded direct contextualization of our finding against prior investigations.

A key finding of our study is that national-level temporal trends of sepsis-related mortality overall and within individual race and ethnicity groups do not reliably represent the corresponding state-level trajectories in terms of both the direction and rate of change over time within each group. This gap between the national and state-level trajectories of sepsis-related mortality limits the inferences that can be gleaned from the former on the evolvement of sepsis epidemiology and on the impact of national efforts to reduce the death toll of sepsis in these populations. We show that even among the Black and Asian groups, which had a national-level decrease in sepsis-related mortality over the last decade, these outcome gains took place only in less than a third of states for each. In addition, the rate of change over time in sepsis-related mortality at the state-level varied widely across states within each race and ethnicity group, varying from as high as nearly 11-fold among White individuals in states with rising mortality to 8-fold among Black individuals for states with decreasing mortality.

States with rising sepsis-related mortality over the past decade within individual race and ethnicity groups represent key priority areas to determine the drivers of these trajectories in order to guide efforts to mitigate the death toll of sepsis. Such examination is also needed for the many states showing lack of improvement in sepsis-related mortality in all races and ethnicity groups. Both of these trajectories are disconcerting given the substantial national-level efforts to date to address the burden of sepsis.

However, there have not been, to our knowledge, contemporary reports on the comparative state-level trends of sepsis-related incidence and case fatality within individual race and ethnicity groups in the US, precluding direct inferences about the relative role of each of these contributors to the state-level trajectories of sepsis-related mortality in our study. Moreover, the optimal approach for trending population-level changes in the incidence of sepsis and thus trends in its case fatality remains unsettled [36]. Importantly, the state-level temporal trends of the exposure within individual race and ethnicity groups to the factors associated with the risk of sepsis (and thus its incidence) and its case fatality were not systematically examined and represent targets for future studies to inform efforts addressing these potentially modifiable factors. However, in contrast to the remaining challenges in estimating reliable trends of the incidence of sepsis and its case fatality, many of the domains associated with these determinants of sepsis-related mortality, especially those at the community and health facility levels, are increasingly tracked in the US across geographical areas, though barriers remain [37].

Many of the aforementioned domains are associated with increased risk of sepsis and its case fatality affect disproportionately racial and ethnic minority groups. Among the domains associated with increased risk of sepsis, Black people have a higher burden of comorbidities [38,39], and alcohol dependence is more common in Black and Hispanic people [40] compared to White people. Residential racial segregation has been long-standing in the US and remains prevalent [41], with greater social deprivation in minority-predominant communities, especially of Black and Hispanic people [42,43]. In addition to its broad association with increased risk of sepsis [10], community-level social deprivation has been shown to be associated with greater prevalence of chronic health conditions, including those associated with sepsis [43,44]. Furthermore, Black people tend to reside more commonly in medically underserved areas [11], with neighborhoods with a high proportion of Black people having less access to primary care [45], and Black and Hispanic people have been increasingly facing barriers to timely medical care compared to White people [46]. Together, the latter disparities can result in delayed diagnosis and inadequate control of comorbid conditions predisposing sepsis and may lead to delayed or inadequate care of infection, which could then progress to sepsis. Indeed, living in medically underserved areas has been associated with increased incidence of sepsis [11].

Once sepsis has developed, greater burden of comorbidities [47] and health behaviors such as alcohol dependence [48], both being noted as more common in some minority groups compared to White people, are associated with increased case fatality. In addition, Black people with sepsis have a higher number of organed dysfunctions compared to White people [49], contributing to increased risk of death among the latter. Community-level social deprivation, which has been affecting disparately racial and ethnic minorities, especially Black and Hispanic populations, is associated with increased case fatality among septic patients, independent of race or ethnicity [15]. Similarly, residing in medically underserved areas is associated with higher case fatality in sepsis, with disparate risk to Black people who reside more often than White people in these areas [11]. Similarly to residential racial segregation, hospital care in the US has been found to be racially segregated, with Black and White people tending to receive care in different hospitals, and hospital segregation has been correlated with residential segregation [50]. Critically, hospital segregation was associated with higher all-cause mortality in Black patients compared to White patients [50], and case fatality of septic patients is higher in hospitals serving predominantly Black and Hispanic communities [24]. These disparities in sepsis outcomes in minority-serving hospitals may be related to overcrowding of emergency departments contributing to delay time to antibiotic therapy, greater strain in intensive care units, less access to subspecialty care and to timely procedures, and patients in these hospitals may have a lower chance for inter-hospital transfer [24]. Together, the greater exposure of racial and ethnic minority groups, and especially of Black people, to the plurality of factors associated with both increased risk of sepsis and its case fatality, compared to White people, places the former at a substantial prognostic disadvantage, not readily addressed by the contemporary national efforts to mitigate the burden of sepsis. Our examination of the inequities in sepsis-related mortality within individual race and ethnicity groups shows that national estimates over the study period obscure substantial and persistent disparities in the death toll of sepsis across states within each group. Although the summary estimates of state-level sepsis-related mortality showed decreased deaths by the end of the last decade among Black and Asian populations, the magnitude of change was relatively small and across-state sepsis-related mortality within race and ethnicity groups varied in 2019 from over 2-fold among Asian individuals to nearly 17-fold among Native American individuals. Our study extends the findings of a prior report by Wang et al., showing substantial across-state disparities in sepsis-related mortality among Black and White individuals, using aggregate data from 1999–2005 [5]. Notably, the states with the highest and lowest sepsis-related mortality rates generally differed across race and ethnicity groups, underscoring the likely variation in the contribution of the factors driving the risk of sepsis and its case fatality across race and ethnicity groups within states and the limitations of one-size-fits-all approaches to reduce the burden of sepsis at both the national and state level.

We noted that in aggregate the gaps in within-state sepsis-related mortality between racial and ethnic minority groups compared to White individuals have decreased among all the examined groups, except among Native American individuals. However, substantial disparities in sepsis-related mortality continued to affect all examined racial and minority groups, with their magnitude varying widely across states. Our study shows that among Black individuals, the gap in within-state sepsis-related mortality compared to White individuals varied over 14-fold across states by the end of the last decade. It is especially disconcerting that the largest Black–White disparity in sepsis-related mortality took place in the region of the national capital, with 80.8 higher age-adjusted sepsis-related mortality rate per 100,000 population than among White individuals. Although the national level sepsis-related mortality rate in 2019 remained lower among Native American individuals compared to Black individuals, the magnitude and variation in within-state disparities among the former was substantially higher, varying 33-fold across states, with the highest gap reaching 96.5 higher age-adjusted sepsis-related mortality rate per 100,000 population than among White individuals in South Dakota.

Nevertheless, considerable changes in the direction of within-state sepsis-related mortality gaps took place in some states in each of the racial and ethnic minority groups, though to varying extents. Thus, although by the end of the last decade the national level sepsis-related mortality rates remained higher among Black and Native American individuals than among White individuals, both groups had lower sepsis-related mortality rates than among White individuals in each three states. A novel finding of this study is that by 2019, sepsis-related mortality rates were lower among Hispanic individuals than among White individuals in 86% of states with available mortality rate data.

However, a substantial source of the decreasing within-state gap in sepsis-related mortality between racial and ethnic minorities and White populations in our study has been the rising sepsis-related mortality among the latter in most states. Thus, when considered in isolation, the apparent decrease in the gap in sepsis-related mortality between racial and ethnic minority groups and White individuals in some states may be erroneously attributed to the decreased disparity, due to the decreased death toll in the minority group. Our findings underscore the importance of considering both the spatial and temporal dimensions of sepsis-related mortality in tracking disparities across race and ethnicity groups.

Thus, together, the trajectories of state-level sepsis-related mortality within individual race and ethnicity groups, changes in disparities of race and ethnicity-specific across-state sepsis-related mortality, and changes in within-state disparities in sepsis-related mortality between racial and ethnic minority groups and White individuals across states represent complementary measures that can be used to refine the tracking of the death toll of sepsis. There are a number of potential uses of these estimates: states and their health departments can use these data to identify priority areas for further examination to tailor policies and determine related fiscal priorities for specific race and ethnicity groups; researchers may use these estimates to determine the corresponding trends in the exposure to the factors underlying the risk of sepsis and its case fatality driving state-specific trajectories of sepsis-related mortality within race and ethnicity groups; physicians could use these data to better understand the health concerns of the populations they serve; and communities can use these estimates as evidence to advocate for change.

The aforementioned lack of validated standards for tracking population-level incidence of sepsis and its case fatality should not delay efforts to explore targeted state-level scalable approaches to address the potentially modifiable drivers of disparities in the risk of sepsis and case fatality among those with sepsis. While no intervention would be expected to result in prompt changes in the trajectories of sepsis-relate mortality, it is crucial that policy makers at the state, local government, and health system levels consistently consider the potentially modifiable factors driving disparities in sepsis-related mortality in policy and fiscal support priorities. The states identified in our study with decreasing sepsis-related mortality within specific racial and ethnic populations can serve as a potential source of policy- and health system-level approaches that may be emulated by states with rising or unchanged sepsis-related mortality trajectories. In line with the evolving paradigm of sepsis as a population- and health system-related health problem [29], the most impactful approaches will likely be those focusing on community- and health system-level domains associated with disparities in the risk of sepsis and its mortality. At the community level, approaches to reverse the prevalent residential segregation of racial and ethnic groups should be explored. Policy efforts, including enforcement of existing fair housing can address the continued discrimination in housing transactions, while reforms to land use regulations could increase access for disadvantaged racial minority groups to well-resourced neighborhoods [51], facilitate broader integration within and across communities in residential developments, and may reduce social deprivation. Efforts to develop and assure safe and available public means of transportation can facilitate in part timely access to primary and specialty care, especially among the older and the disabled who are at increased risk of sepsis and its case fatality in minority-predominant communities.

At the health system level, the current segregation in access to primary care in urban minority-predominant areas would benefit from a fresh state-level re-examination of incentives for primary care clinicians to practice in these communities. The recent introduction of the Hospital Sepsis Program Core Elements by the CDC, which aims to provide hospitals with a “a manager’s guide” to develop comprehensive programs to monitor and improve outcomes of sepsis [52], is a step in the right direction in the efforts to mitigate the burden of sepsis. However, together with the recent change in the Centers for Medicare and Medicaid Services Severe Sepsis/Septic Shock Early Management Bundle (SEP-1) from pay-for-reporting to pay-for-performance in 2024 [53], these initiatives may pose substantial challenges to hospitals caring predominantly for Black and Hispanic patients. These hospitals, where patients with sepsis have been shown to have higher case fatality compared to those managed in nonminority hospitals [24], are generally more strained financially, often struggle to maintain adequate staffing, and are more likely be penalized financially for their inability to adequately comply. Efforts to reduce variation in sepsis-related case fatality across hospitals should examine policies to provide more sustainable fiscal support to minority-serving hospitals, examine incentives to improve staffing in struggling facilities, and expand health insurance coverage to minority communities, which may help reduce segregation in hospital care and possibly facilitate inter-hospital transfer of septic patients when needed. Finally, accelerating state- and national-level efforts to create a common framework to unify the currently numerous standards for data generation, storage, access, mobility, and security, in order to facilitate effective tracking of the now ubiquitous patient-, community-, and health system-level data is needed to allow for the tracking of the impact of efforts to reduce disparities in exposure to the drivers of the risk of sepsis and its case fatality, and to decrease their overall prevalence [36,54].

### Study limitations

This study has several limitations in addition to those noted earlier. First, the accuracy of death certificate diagnoses could not be directly verified and misclassification in listed causes of death from errors in diagnosis and the completion of death certificates, as well as miscoding, could have occurred. Second, the designation of race and ethnicity in death certificates is often based on personal observations of funeral directors and may lead to misclassification [55]. There have been, however, no reports, to our knowledge, on systematic state-level changes over time in the race and ethnicity designations in death certificates within individual race and ethnicity groups overall and for diagnoses of sepsis. Third, suppression of death data and limitations in deriving mortality rates for low death counts in the CDC WONDER data set, together with the heterogeneity of racial and ethnic minority populations across the country, limited the number of states with mortality rate estimates among racial and ethnic minority groups, affecting predominantly Native American individuals. Notably, Native American, as well as Asian individuals, were often excluded from race-specific analyses in epidemiological studies of sepsis [2,5,6,12], likely in part due to concerns about their small numbers and related statistical constraints. However, our findings that Native American individuals had some of the highest state-level disparities in sepsis-related mortality underscore the importance of an inclusive examination of the toll of sepsis across vulnerable populations, and the public health challenges of efforts to reduce the burden of sepsis in regionally small populations of underrepresented racial and ethnic minority groups. Fourth, the population data in our study were based in part on intercensal estimates that may be subject to error. However, there have not been, to our knowledge, reports indicating systematic errors in population estimates within individual races and ethnicity groups nationally or at the state level. Fifth, although we used an ICD-10 code-based taxonomy employed in prior studies of sepsis-related mortality in the US, based on CDC WONDER data, to identify decedents with sepsis, it has not been validated and the optimal approach to identify sepsis in aggregated death certificate-based data repositories remains unknown. Sixth, the introduction of the Sepsis-3 consensus definitions in early 2016 could have affected trends in sepsis-related mortality starting that year. However, there was no discontinuity in sepsis-related mortality rates for the US population between the years 2015 and 2016, and similar changes in trends of sepsis-related mortality were demonstrated, whether estimated by comparing the time period prior to 2016 to that starting on 2016, or when comparing the time period prior to 2015 to that starting 2015, thus not supporting a distinct impact on mortality trends starting specifically in 2016. Our modeling approach has been similar to that of Prest el al, who examined sepsis-related mortality trends in the US during 2005–2018 [4]. Nevertheless, we cannot completely discount a potential impact of the introduction of the Sepsis-3 definitions on tracked sepsis epidemiology. Seventh, our data did not include the COVID-19 pandemic and our findings may not be generalizable to that period. COVID-19-related organ dysfunction is increasingly considered to meet the Sepsis-3 framework [56], though the extent and validity of the diagnosis of COVID-19-related sepsis in clinical practice and in death certificates at the national and state levels over the pandemic period remain unknown. Last, our findings may not be generalizable to other geographic areas and health systems. Nearly 75% of sepsis deaths worldwide in 2017 took place in low- and middle-income countries [1], but these areas often face substantial challenges in trending national and regional trajectories of sepsis-related mortality overall and in specific racial strata. At the same time, the substantial and accelerating migration from these areas to high-income countries may amplify outcome disparities in these regions. However, while more feasible in high-income countries, national and regional data on the race- and ethnicity-specific trajectories of sepsis-related mortality are lacking, thus limiting inferences on the evolving epidemiology of sepsis and on the race and ethnicity-specific impact of national efforts to curb the death toll of sepsis in these areas. Our findings suggest, however, the need for a similar exploration in other countries and health systems.

## 5. Conclusions

National-level estimates of changes in sepsis-related mortality did not reflect state-level trajectories. There was substantial state-level variation in the direction of the trajectories of sepsis-related mortality in the US among all examined race and ethnicity groups, while national-level outcome gains among racial groups were not shared equitably across states. Substantial disparities in sepsis-related mortality across states persisted within all race and ethnicity groups, as did within-state disparities in sepsis-related mortality between racial and ethnic minority groups and White individuals.

## Figures and Tables

**Table 1:** Trends of national sepsis-related mortality in the United States, 2010–2019.

	2010		2019			
Group	Deaths	AAMR ^a^ (95% CI) ^b^	Deaths	AAMR (95% CI)	APC ^c,d^ (95% CI)	*p* Value ^d^
**All**	159,982	48.4 (48.1–48.6)	201,478	49.8 (49.6–50.0)	+0.9 (+0.4 to +1.5)	**0.0013**
**Race and ethnicity**						
Black	24,587	79.5 (78.5–80.5)	29,416	73.0 (72.1–73.9)	**−0.7 (−1.1 to −0.2)**	**0.0043**
Hispanic	12,217	48.9 (48.0–49.9)	18,074	45.2 (44.5–45.9)	−0.2 (−0.7 to +0.3)	0.3691
Asian	4006	34.3 (33.2–35.4)	6247	30.9 (30.1–31.7)	**−0.7 (−1.1 to −0.2)**	**0.0039**
Native American	1187	51.2 (48.0–54.3)	1705	65.5 (62.3–68.7)	+0.5 (−0.4 to +1.5)	0.2762
White	117,985	45.5 (45.3–45.8)	146,034	48.0 (47.2–48.7)	**+1.4 (+0.8 to +2.0)**	**<0.0001**

^a^ AAMR: age-adjusted mortality rates, expressed per 100,000 population; ^b^ 95% CI: 95% confidence interval; ^c^ APC: annual percent change; ^d^ boldfaced entries represent statistically significant trends.

## Data Availability

The original data presented in the study are openly available in the Centers for Disease Control and Prevention Wide-ranging Online Data for Epidemiological Research (CDC WONDER) database, accessible at https://wonder.cdc.gov/mcd.html (accessed on 23 September 2023).

## Supplementary Materials

The following supporting information can be downloaded at: https://www.mdpi.com/article/10.3390/jcm13102848/s1, Table S1. Trends of state-level sepsis-related mortality overall, 2010–2019. Table S2. Trends of state-level sepsis-related mortality among Black individuals, 2010–2019. Table S3. Trends of state-level sepsis-related mortality among Hispanic individuals, 2010–2019. Table S4. Trends of state-level sepsis-related mortality among Asian individuals, 2010–2019. Table S5. Trends of state-level sepsis-related mortality among Native American individuals, 2010–2019. Table S6. Trends of state-level sepsis-related mortality among White individuals, 2010–2019. Table S7. Changes in age-adjusted sepsis-related mortality rates nationally and across states in 2010 vs. 2019. Table S8. Changes in differences in within-state age-adjusted sepsis-related mortality rates between racial and ethnic minority groups and White individuals, nationally and across states in 2010 vs. 2019. Table S9. Individual state data of differences in within-state age-adjusted sepsis-related mortality rates—Black vs. White individuals, 2010 vs. 2019. Table S10. Individual state data of differences in within-state age-adjusted sepsis-related mortality rates—Hispanic vs. White individuals, 2010 vs. 2019. Table S11. Individual state data of differences in within-state age-adjusted sepsis-related mortality rates—Asian vs. White individuals, 2010 vs. 2019. Table S12. Individual state data of differences in within-state age-adjusted sepsis-related mortality rates—Native American vs. White individuals, 2010 vs. 2019. Table S13. Interrupted time series analysis of changes in sepsis-related mortality for the United States population, 2010–2019 (comparison of the 2010–2015 and 2016–2019 periods). Table S14. Interrupted time series analysis of changes in sepsis-related mortality for the United States population, 2010–2019 (comparison of the 2010–2014 and 2015–2019 periods).
