# Supplementary material for: Trajectories of State-Level Sepsis-Related Mortality by Race and Ethnicity Group in the United States
Source: J Clin Med. 2024 May 12;13(10):2848. doi: 10.3390/jcm13102848 (PMC11122657; doi:10.3390/jcm13102848)
Supplement: Supplementary file 1 [file jcm-13-02848-s001.zip › jcm-2946345-supplementary.pdf]

**Trajectories of State-Level Sepsis-Related Mortality by Race and Ethnicity Group in the United States**

**Lavi Oud, M.D., John Garza, Ph.D.**

**Supplementary Data**

**Table of contents:**

**Table S1. Trends of state-level sepsis-related mortality, overall, 2010-2019**

**Table S2. Trends of state-level sepsis-related mortality among Black individuals, 2010-2019**

**Table S3. Trends of state-level sepsis-related mortality among Hispanic individuals, 2010-2019**

**Table S4. Trends of state-level sepsis-related mortality among Asian individuals, 2010-2019**

**Table S5. Trends of state-level sepsis-related mortality among Native American individuals, 2010-2019**

**Table S6. Trends of state-level sepsis-related mortality among White individuals, 2010-2019**

**Table S7. Changes in age-adjusted sepsis-related mortality rates nationally and across states in 2010 vs 2019**

**Table S8. Changes in differences in within-state age-adjusted sepsis-related mortality rates between racial and ethnic minority groups and White individuals, nationally and across states in 2010 vs 2019**

**Table S9. Individual state data of differences in within-state age-adjusted sepsis-related mortality rates – Black vs White individuals, 2010 vs 2019**

**Table S10. Individual state data of differences in within-state age-adjusted sepsis-related mortality rates – Hispanic vs White individuals, 2010 vs 2019**

**Table S11. Individual state data of differences in within-state age-adjusted sepsis-related mortality rates – Asian vs White individuals, 2010 vs 2019**

**Table S12. Individual state data of differences in within-state age-adjusted sepsis-related mortality rates – Native American vs White individuals, 2010 vs 2019**

**Table S13. Interrupted time series analysis of changes in sepsis-related mortality for the United States population, 2010-2019 (comparison of the 2010-2015 and 2016-2019 periods)**

**Table S14. Interrupted time series analysis of changes in sepsis-related mortality for the United States population, 2010-2019 (comparison of the 2010-2014 and 2015-2019 periods)**

**Table S1. Trends of state-level sepsis-related mortality, overall, 2010-2019**

| State | 2010 | | 2019 | | | |
|---|---|---|---|---|---|---|
| | **Deaths** | **AAMR[a] (95% CI)[b]** | **Deaths** | **AAMR (95% CI)** | **APC[c,d] (95% CI)** | **p value[d]** |
| Alabama | 3043 | 58.8 (56.7 - 60.9) | 3920 | 63.3 (61.3 - 65.3) | **+1.5 (+1.0 to +2.0)** | **<0.0001** |
| Alaska | 179 | 37.4 (31.4 - 43.4) | 290 | 45.9 (40.4 - 51.5) | **+2.1 (+0.8 to +3.4)** | **0.0017** |
| Arizona | 2669 | 39.2 (37.7 - 40.7) | 2409 | 26.2 (25.1 - 27.3) | **-2.7 (-5.4 to 0.0)** | **0.0488** |
| Arkansas | 1760 | 54.0 (51.5 - 56.6) | 2624 | 68.4 (65.8 - 71.1) | **+3.1 (+2.2 to +3.9)** | **<0.0001** |
| California | 17437 | 48.4 (47.7 - 49.1) | 21919 | 48.7 (48.0 - 49.4) | **+0.6 (+0.1 to +1.1)** | **0.0245** |
| Colorado | 1499 | 32.5 (30.9 - 34.2) | 2430 | 39.1 (37.5 - 40.7) | **+3.4 (+2.3 to +4.6)** | **<0.0001** |
| Connecticut | 2130 | 48.7 (46.6 - 50.8) | 2564 | 51.7 (49.6 - 53.7) | **+0.8 (+0.2 to +1.3)** | **0.0038** |
| Delaware | 504 | 49.6 (45.2 - 53.9) | 535 | 40.5 (37.0 - 44.0) | 0.0 (-2.4 to +2.4) | 0.9723 |
| District of Columbia | 484 | 84.4 (76.8 - 92.0) | 478 | 68.9 (62.6 - 75.2) | **-2.0 (-3.7 to -0.3)** | **0.0206** |
| Florida | 9059 | 36.2 (35.4 - 37.0) | 13667 | 42.2 (41.5 - 43.0) | **+2.3 (+1.8 to +2.8)** | **<0.0001** |
| Georgia | 5113 | 60.4 (58.7 - 62.1) | 6781 | 59.5 (58.0 - 60.9) | +0.4 (-0.1 to +0.8) | 0.0936 |
| Hawaii | 587 | 36.1 (33.1 - 39.0) | 563 | 29.3 (26.8 - 31.8) | **-1.6 (-2.7 to -0.4)** | **0.0077** |
| Idaho | 479 | 30.3 (27.6 - 33.1) | 807 | 39.0 (36.3 - 41.7) | **+2.5 (+1.6 to +3.4)** | **<0.0001** |
| Illinois | 7162 | 53.1 (51.9 - 54.3) | 7888 | 50.4 (49.3 - 51.5) | -0.2 (-0.7 to +0.4) | 0.5667 |
| Indiana | 3319 | 47.9 (46.2 - 49.5) | 4801 | 59.3 (57.6 - 61.0) | **+2.4 (+1.8 to +3.0)** | **<0.0001** |
| Iowa | 1211 | 31.8 (30.0 - 33.6) | 1817 | 43.3 (41.2 - 45.3) | **+4.2 (+3.2 to +5.1)** | **<0.0001** |
| Kansas | 1191 | 37.6 (35.4 - 39.7) | 1891 | 51.9 (49.5 - 54.3) | **+4.3 (+3.2 to +5.4)** | **<0.0001** |

| | | | | | | |
|---|---|---|---|---|---|---|
| Kentucky | 2963 | 63.9 (61.6 - 66.2) | 4575 | 83.2 (80.8 - 85.7) | **+4.2 (+3.0 to +5.4)** | **<0.0001** |
| Louisiana | 2895 | 64.3 (61.9 - 66.7) | 3286 | 59.9 (57.9 - 62.0) | +0.2 (-0.6 to +0.9) | 0.6750 |
| Maine | 625 | 36.8 (33.8 - 39.7) | 437 | 22.5 (20.3 - 24.7) | -2.6 (-5.3 to +0.3) | 0.0752 |
| Maryland | 3310 | 55.7 (53.8 - 57.6) | 3618 | 49.4 (47.8 - 51.1) | **-1.2 (-1.9 to -0.6)** | **0.0002** |
| Massachusetts | 3405 | 44.3 (42.8 - 45.8) | 4177 | 46.2 (44.7 - 47.6) | **+1.3 (+0.4 to +2.2)** | **0.0063** |
| Michigan | 5548 | 49.4 (48.1 - 50.7) | 6368 | 48.7 (47.5 - 49.9) | -0.1 (-0.6 to +0.3) | 0.5210 |
| Minnesota | 1584 | 27.2 (25.9 - 28.6) | 2466 | 35.2 (33.7 - 36.6) | **+3.6 (+2.7 to +4.4)** | **<0.0001** |
| Mississippi | 1980 | 65.6 (62.6 - 68.5) | 2708 | 76.4 (73.5 - 79.4) | **+2.4 (+1.2 to +3.5)** | **<0.0001** |
| Missouri | 2989 | 44.1 (42.5 - 45.7) | 3701 | 46.7 (45.2 - 48.2) | **+0.8 (0.0 to +1.5)** | **0.0426** |
| Montana | 352 | 30.0 (26.8 - 33.2) | 589 | 41.3 (37.9 - 44.8) | **+4.9 (+3.5 to +6.2)** | **<0.0001** |
| Nebraska | 634 | 30.6 (28.2 - 33.0) | 1035 | 43.7 (40.9 - 46.4) | **+5.1 (+4.2 to +6.1)** | **<0.0001** |
| Nevada | 1419 | 56.4 (53.4 - 59.4) | 1821 | 50.9 (48.5 - 53.2) | +0.4 (-1.0 to +1.8) | 0.5795 |
| New Hampshire | 528 | 36.0 (32.9 - 39.2) | 748 | 40.5 (37.5 - 43.4) | **+1.5 (+0.6 to +2.5)** | **0.0018** |
| New Jersey | 6314 | 63.2 (61.6 - 64.7) | 6546 | 57.1 (55.7 - 58.5) | **-0.8 (-1.2 to -0.5)** | **<0.0001** |
| New Mexico | 970 | 45.0 (42.1 - 47.8) | 1311 | 50.2 (47.4 - 53.0) | **+1.4 (+0.7 to +2.0)** | **0.0001** |
| New York | 9833 | 44.8 (43.9 - 45.7) | 10821 | 42.4 (41.6 - 43.2) | -0.5 (-1.5 to +0.6) | 0.3915 |
| North Carolina | 5468 | 55.7 (54.2 - 57.2) | 6659 | 52.2 (50.9 - 53.4) | +0.1 (-0.8 to +1.0) | 0.8218 |
| North Dakota | 273 | 32.6 (28.7 - 36.6) | 442 | 47.6 (43.1 - 52.2) | **+3.7 (+2.4 to +5.1)** | **<0.0001** |
| Ohio | 6770 | 50.7 (49.5 - 51.9) | 7490 | 49.2 (48.1 - 50.3) | +0.1 (-0.5 to +0.7) | 0.7288 |
| Oklahoma | 2398 | 59.6 (57.2 - 62.0) | 3403 | 72.2 (69.8 - 74.7) | **+3.2 (+2.3 to +4.0)** | **<0.0001** |
| Oregon | 1374 | 30.9 (29.3 - 32.6) | 1844 | 34.0 (32.4 - 35.5) | **+1.8 (+0.8 to +2.8)** | **0.0005** |
| Pennsylvania | 7889 | 48.6 (47.6 - 49.7) | 8724 | 48.3 (47.3 - 49.3) | +0.4 (-0.1 to +1.0) | 0.1259 |
| Rhode Island | 729 | 55.5 (51.4 - 59.6) | 631 | 43.5 (40.0 - 46.9) | **-2.7 (-3.9 to -1.5)** | **<0.0001** |
| South Carolina | 2969 | 60.3 (58.1 - 62.5) | 4193 | 64.0 (62.0 - 66.0) | **+1.6 (+0.6 to +2.6)** | **0.0019** |
| South Dakota | 296 | 29.9 (26.4 - 33.4) | 634 | 56.4 (51.8 - 60.9) | **+6.8 (+5.5 to +8.1)** | **<0.0001** |
| Tennessee | 3887 | 57.4 (55.6 - 59.2) | 5341 | 63.8 (62.1 - 65.6) | **+2.0 (+1.4 to +2.5)** | **<0.0001** |
| Texas | 13037 | 60.2 (59.2 - 61.3) | 17091 | 59.6 (58.7 - 60.5) | +0.7 (-0.1 to +1.6) | 0.1042 |
| Utah | 654 | 31.0 (28.6 - 33.5) | 1136 | 41.1 (38.6 - 43.5) | **+3.0 (+1.5 to +4.5)** | **0.0001** |
| Vermont | 254 | 34.0 (29.8 - 38.3) | 272 | 30.4 (26.7 - 34.1) | **-1.8 (-3.3 to -0.3)** | **0.0165** |
| Virginia | 4313 | 54.3 (52.7 - 56.0) | 4624 | 45.4 (44.1 - 46.8) | **-1.3 (-2.4 to -0.2)** | **0.0243** |

| | | | | | | |
|---|---|---|---|---|---|---|
| Washington | 2787 | 40.1 (38.6 - 41.6) | 4020 | 45.3 (43.9 - 46.7) | **+1.7 (+1.0 to +2.4)** | **<0.0001** |
| West Virginia | 1421 | 61.2 (57.9 - 64.4) | 2055 | 80.8 (77.1 - 84.4) | **+3.6 (+3.0 to +4.2)** | **<0.0001** |
| Wisconsin | 2090 | 32.0 (30.6 - 33.4) | 3064 | 40.4 (38.9 - 41.8) | **+3.6 (+2.6 to +4.6)** | **<0.0001** |
| Wyoming | 199 | 34.2 (29.4 - 39.0) | 264 | 37.6 (33.0 - 42.3) | **+4.3 (+1.8 to +6.9)** | **0.0007** |

a AAMR: age-adjusted mortality rates, expressed per 100,000 population

b 95% CI: 95% confidence interval

c APC: Annual Percent Change

d Boldfaced entries represent statistically significant trends

**Table S2. Trends of state-level sepsis-related mortality among Black individuals, 2010-2019**

| State | 2010 | | 2019 | | | |
|---|---|---|---|---|---|---|
| | **Deaths** | **AAMR[a] (95% CI)[b]** | **Deaths** | **AAMR (95% CI)** | **APC[c,d] (95% CI)** | **p value[d]** |
| Alabama | 871 | 83.5 (77.8-89.2) | 1077 | 80.1 (75.2 - 85.0) | +0.4 (-0.5 to +1.4) | 0.3604 |
| Arizona | 100 | 55.5 (43.7-67.4) | 99 | 32.7 (26.3 - 40.3) | **-4.1 (-7.4 to -0.6)** | **0.0209** |
| Arkansas | 297 | 84.0 (74.1-93.8) | 443 | 102.0 (92.1 - 111.9) | **+2.8 (+1.7 to +3.9)** | **<0.0001** |
| California | 1589 | 76.6 (72.7-80.4) | 1965 | 72.9 (69.6 - 76.2) | -0.1 (-0.7 to +0.6) | 0.8781 |
| Colorado | 68 | 49.9 (38.1-64.2) | 125 | 60.7 (49.5 - 71.9) | **+2.9 (+1.0 to +4.9)** | **0.0023** |
| Connecticut | 184 | 64.7 (55.0-74.4) | 237 | 61.6 (53.6 - 69.6) | -0.1 (-1.6 to +1.6) | 0.9803 |
| Delaware | 91 | 57.1 (45.5-70.8) | 128 | 55.9 (45.9 - 65.8) | +0.8 (-2.1 to +3.8) | 0.5902 |
| District of Columbia | 418 | 116.8 (105.5-128.1) | 412 | 104.0 (93.7 - 114.2) | -1.2 (-3.3 to +0.9) | 0.2747 |
| Florida | 1322 | 58.3 (55.1-61.6) | 1932 | 56.8 (54.2 - 59.4) | +0.6 (-0.4 to +1.5) | 0.2368 |
| Georgia | 1775 | 90.3 (85.8 - 94.7) | 2243 | 75.6 (72.3 - 78.9) | **-1.3 (-2.0 to -0.6)** | **0.0005** |
| Illinois | 1534 | 98.6 (93.5 - 103.6) | 1478 | 76.5 (72.5 - 80.5) | **-2.5 (-3.4 to -1.7)** | **<0.0001** |
| Indiana | 349 | 77.8 (69.4 - 86.3) | 414 | 74.0 (66.6 - 81.3) | -0.2 (-1.1 to +0.7) | 0.6799 |
| Iowa | 27 | 56.7 (36.0 - 85.1) | 39 | 58.8 (40.4 - 82.5) | +1.3 (-1.6 to +4.4) | 0.3814 |

| | | | | | | |
|---|---|---|---|---|---|---|
| Kansas | 71 | 59.0 (45.7 - 74.9) | 120 | 78.6 (64.1 - 93.2) | **+3.3 (+1.2 to +5.4)** | **0.0016** |
| Kentucky | 237 | 90.8 (78.9 - 102.8) | 316 | 91.4 (80.9 - 101.8) | **+1.8 (+0.1 to +3.7)** | **0.0410** |
| Louisiana | 1014 | 91.3 (85.5 - 97.1) | 1170 | 80.9 (76.1 - 85.7) | **-0.8 (-1.4 to -0.2)** | **0.0086** |
| Maryland | 1165 | 87.4 (82.2 - 92.6) | 1272 | 66.8 (63.0 - 70.5) | **-2.3 (-3.3 to -1.3)** | **<0.0001** |
| Massachusetts | 165 | 47.4 (39.9 - 54.9) | 231 | 43.4 (37.6 - 49.1) | -0.1 (-1.5 to +1.4) | 0.9514 |
| Michigan | 1187 | 98.9 (93.1 - 104.6) | 1219 | 84.5 (79.7 - 89.4) | **-2.0 (-2.8 to -1.2)** | **<0.0001** |
| Minnesota | 43 | 31.9 (22.0 - 44.9) | 98 | 45.9 (36.3 - 57.3) | **+2.5 (+0.7 to +4.3)** | **0.0068** |
| Mississippi | 790 | 96.2 (89.3 - 103.1) | 1005 | 97.0 (90.8 - 103.2) | +0.9 (-0.2 to +2.1) | 0.1237 |
| Missouri | 411 | 76.4 (68.9 - 84.0) | 490 | 70.4 (64.0 - 76.8) | +0.1 (-1.1 to +1.2) | 0.9384 |
| Nebraska | 27 | 53.5 (34.3 - 79.6) | 35 | 49.2 (33.7 - 69.5) | +0.2 (+3.6 to +4.1) | 0.9129 |
| Nevada | 124 | 73.3 (59.4 - 87.3) | 177 | 62.9 (53.3 - 72.6) | -0.3 (-1.9 to +1.2) | 0.6632 |
| New Jersey | 1076 | 105.6 (99.1 - 112.1) | 1094 | 81.5 (76.6 - 86.4) | **-2.4 (-3.2 to -1.7)** | **<0.0001** |
| New Mexico | 22 | 47.7 (29.5-72.8) | 20 | 40.3 (24.3 - 62.9) | +2.9 (-7.7 to +14.7) | 0.6061 |
| New York | 1390 | 47.8 (45.2 - 50.3) | 1832 | 48.7 (46.4 - 50.9) | +0.3 (-1.3 to +1.9) | 0.7323 |
| North Carolina | 1334 | 79.4 (75.0 - 83.8) | 1611 | 68.9 (65.4 - 72.4) | **-1.1 (-2.0 to -0.3)** | **0.0094** |
| Ohio | 1014 | 82.8 (77.6 - 88.0) | 949 | 62.4 (58.3 - 66.4) | **-2.5 (-3.2 to -1.8)** | **<0.0001** |
| Oklahoma | 186 | 89.8 (76.4 - 103.3) | 219 | 80.1 (69.1 - 91.2) | +0.8 (-1.1 to +2.8) | 0.4054 |
| Oregon | 24 | 52.6 (32.6 - 80.4) | 37 | 49.2 (33.9 - 69.1) | -1.7 (-4.8 to +1.3) | 0.2620 |
| Pennsylvania | 969 | 79.9 (74.8 - 85.0) | 1035 | 70.2 (65.9 - 74.6) | **-1.3 (-2.2 to -0.5)** | **0.0014** |
| Rhode Island | 27 | 52.2 (33.5 - 77.7) | 25 | 34.7 (22.0 - 52.1) | **-6.4 (-10.3 to -2.3)** | **0.0023** |
| South Carolina | 1004 | 92.5 (86.6 - 98.4) | 1305 | 90.7 (85.7 - 95.8) | +0.5 (-0.1 to +1.1) | 0.1239 |
| Tennessee | 657 | 83.6 (76.9 - 90.3) | 902 | 87.4 (81.4 - 93.3) | **+1.0 (+0.4 to +1.8)** | **0.0031** |
| Texas | 1871 | 89.9 (85.6 - 94.2) | 2394 | 80.1 (76.7 - 83.4) | -0.3 (-1.4 to +0.8) | 0.6183 |
| Virginia | 1007 | 78.0 (73.1 - 83.0) | 1072 | 61.9 (58.1 - 65.7) | **-2.1 (-3.3 to -1.0)** | **0.0003** |
| Washington | 96 | 61.8 (49.1 - 76.8) | 151 | 61.9 (51.2 - 72.6) | +1.5 (-1.3 to +4.4) | 0.3053 |

| | | | | | | |
|---|---|---|---|---|---|---|
| West Virginia | 43 | 67.6 (48.5 - 91.7) | 66 | 90.0 (69.0 - 115.4) | +2.1 (-0.6 to +4.8) | 0.1253 |
| Wisconsin | 147 | 69.0 (57.0 - 81.1) | 185 | 62.9 (53.3 - 72.4) | +0.1 (-1.9 to +2.1) | 0.9492 |

a AAMR: age-adjusted mortality rates, expressed per 100,000 population

b 95% CI: 95% confidence interval

c APC: Annual Percent Change

d Boldfaced entries represent statistically significant trends

**Table S3. Trends of state-level sepsis-related mortality among Hispanic individuals, 2010-2019**

| State | 2010 | | 2019 | | | |
|---|---|---|---|---|---|---|
| | **Deaths** | **AAMR[a] (95% CI)[b]** | **Deaths** | **AAMR (95% CI)** | **APC[c,d] (95% CI)** | **p value[d]** |
| Arizona | 381 | 42.9 (38.2 - 47.6) | 420 | 27.3 (24.6 - 30.0) | -2.3 (-5.4 to +0.9) | 0.1622 |
| Arkansas | 21 | 39.3 (21.5-65.9) | 29 | 32.3 (20.2 - 48.9) | -0.4 (-6.1 to +5.6) | 0.8853 |
| California | 3632 | 52.5 (50.7 - 54.3) | 5313 | 50.7 (49.2 - 52.1) | +0.1 (-0.5 to +0.6) | 0.8657 |
| Colorado | 204 | 39.7 (33.9 - 45.6) | 352 | 44.8 (39.9 - 49.8) | **+2.8 (+1.0 to +4.6)** | **0.0021** |
| Connecticut | 110 | 52.2 (41.3 - 63.0) | 160 | 45.9 (38.4 - 53.4) | +0.7 (-0.8 to +2.3) | 0.3584 |
| Florida | 1143 | 33.2 (31.3 - 35.1) | 1905 | 33.7 (32.2 - 35.2) | +0.6 (-0.2 to +1.3) | 0.1406 |
| Georgia | 56 | 24.3 (17.1 - 33.6) | 142 | 31.2 (25.4 - 37.0) | **+5.5 (+0.9 to +10.3)** | **0.0196** |
| Idaho | 23 | 35.0 (20.4-56.0) | 42 | 42.6 (29.7 - 59.3) | +3.4 (-2.6 to +9.7) | 0.2744 |
| Illinois | 380 | 47.9 (42.5 - 53.3) | 523 | 40.2 (36.6 - 43.9) | **-3.1 (-4.3 to -1.9)** | **<0.0001** |
| Indiana | 60 | 49.1 (36.3 - 64.9) | 95 | 44.1 (35.0 - 54.7) | +0.3 (-2.6 to +3.3) | 0.8536 |
| Kansas | 37 | 37.8 (25.7 - 53.6) | 72 | 42.8 (32.9 - 54.8) | +1.1 (-2.8 to +5.2) | 0.5776 |
| Louisiana | 28 | 27.4 (17.8 - 40.5) | 40 | 23.1 (16.3 - 31.7) | +2.1 (-0.9 to +5.2) | 0.1770 |
| Maryland | 49 | 21.9 (15.2 - 30.6) | 90 | 28.5 (22.4 - 35.8) | +0.7 (-1.6 to +3.1) | 0.5469 |
| Massachusetts | 94 | 32.9 (25.8 - 41.4) | 177 | 36.6 (30.9 - 42.3) | +1.5 (-0.6 to +3.6) | 0.1741 |
| Michigan | 92 | 48.5 (38.3 - 60.5) | 118 | 39.3 (31.8 - 46.7) | -0.7 (-2.1 to +0.8) | 0.3523 |

| | | | | | | |
|---|---|---|---|---|---|---|
| Minnesota | 22 | 32.1 (18.0 - 53.0) | 38 | 32.7 (22.1 - 46.7) | -2.9 (-7.0 to +1.5) | 0.1942 |
| Missouri | 28 | 25.8 (16.5-38.4) | 35 | 21.9 (14.8 - 31.2) | +0.1 (-5.2 to +5.5) | 0.9941 |
| Nebraska | 27 | 43.1 (26.7-65.9) | 38 | 39.3 (26.5 - 56.0) | -2.4 (-7.4 to +2.8) | 0.3526 |
| Nevada | 124 | 43.7 (34.6 - 52.7) | 182 | 35.9 (30.3 - 41.5) | -1.5 (-3.0 to +0.1) | 0.0548 |
| New Jersey | 520 | 67.4 (61.2 - 73.5) | 656 | 49.9 (46.0 - 53.9) | **-2.4 (-3.3 to -1.5)** | **<0.0001** |
| New Mexico | 349 | 48.5 (43.3 - 53.7) | 460 | 46.9 (42.5 - 51.2) | +0.8 (-0.2 to +1.8) | 0.1149 |
| New York | 794 | 38.1 (35.4 - 40.9) | 1103 | 35.6 (33.5 - 37.7) | -3.7 (-6.0 to -1.3) | 0.4393 |
| North Carolina | 47 | 25.1 (16.9 - 35.8) | 95 | 24.5 (19.2 - 30.9) | +2.5 (-0.2 to +5.2) | 0.0663 |
| Ohio | 57 | 39.6 (29.4 - 52.1) | 78 | 30.2 (23.5 - 38.2) | **-2.3 (-4.1 to -0.5)** | **0.0113** |
| Oklahoma | 33 | 33.5 (21.9 - 49.1) | 99 | 57.3 (45.5 - 71.2) | **+5.7 (+3.1 to +8.5)** | **<0.0001** |
| Oregon | 36 | 27.4 (18.0 - 39.8) | 66 | 26.5 (19.9 - 34.7) | **+3.1 (+0.5 to +5.7)** | **0.0181** |
| Pennsylvania | 153 | 50.0 (41.2 - 58.8) | 198 | 36.8 (31.4 - 42.3) | -0.8 (-2.8 to +1.2) | 0.4253 |
| Rhode Island | 25 | 48.4 (29.2 - 75.6) | 32 | 31.7 (21.1 - 45.8) | **-5.1 (-9.1 to -1.0)** | **0.0164** |
| South Carolina | 21 | 25.5 (13.6 - 43.6) | 41 | 28.0 (19.2 - 39.3) | +1.2 (-2.9 to +5.6) | 0.5676 |
| Tennessee | 21 | 26.4 (14.4 - 44.2) | 54 | 31.1 (22.4 - 42.0) | +0.7 (-1.7 to +3.1) | 0.5720 |
| Texas | 3373 | 70.8 (68.3 - 73.4) | 4833 | 65.3 (63.3 - 67.2) | -0.2 (-0.8 to +0.3) | 0.4489 |
| Utah | 30 | 27.7 (17.6 - 41.6) | 79 | 35.8 (27.5 - 45.8) | +3.9 (-0.6 to +8.6) | 0.0920 |
| Virginia | 60 | 36.4 (26.9 - 48.3) | 93 | 23.2 (18.2 - 29.1) | -1.8 (-4.4 to -0.8) | 0.1734 |
| Washington | 73 | 27.0 (19.8 - 36.0) | 159 | 40.6 (33.5 - 47.6) | **+4.3 (+2.0 to +6.7)** | **0.0002** |
| Wisconsin | 35 | 32.7 (21.2 - 48.3) | 63 | 32.9 (24.6 - 43.2) | +1.5 (-1.2 to +4.3) | 0.2731 |

a AAMR: age-adjusted mortality rates, expressed per 100,000 population

b 95% CI: 95% confidence interval

c APC: Annual Percent Change

d Boldfaced entries represent statistically significant trends

**Table S4. Trends of state-level sepsis-related mortality among Asian individuals, 2010-2019**

| State | 2010 | | 2019 | | | |
|---|---|---|---|---|---|---|
| | **Deaths** | **AAMR[a] (95% CI)[b]** | **Deaths** | **AAMR (95% CI)** | **APC[c,d] (95% CI)** | **p value[d]** |
| Arizona | 28 | 20.5 (13.1 - 30.5) | 53 | 19.6 (14.5 - 25.8) | -1.9 (-5.0 to +1.4) | 0.2527 |
| California | 1843 | 38.6 (36.8 - 40.3) | 2662 | 34.2 (32.9 - 35.6) | **-0.6 (-1.2 to -0.1)** | **0.0176** |
| Colorado | 23 | 26.1 (16.1 - 39.8) | 41 | 22.4 (16.0 - 30.7) | +1.1 (-2.2 to +4.4) | 0.5252 |
| Connecticut | 21 | 36.7 (21.4 - 58.8) | 35 | 25.9 (17.7 - 36.6) | **-3.6 (-6.7 to -0.4)** | **0.0262** |
| Florida | 87 | 23.0 (18.1 - 29.0) | 166 | 22.8 (19.3 - 26.3) | +1.6 (-0.2 to +3.7) | 0.0767 |
| Georgia | 54 | 35.1 (25.2 - 47.6) | 117 | 32.2 (26.0 - 38.4) | +0.5 (-1.4 to +2.4) | 0.6043 |
| Hawaii | 442 | 37.5 (33.9 - 41.0) | 438 | 32.4 (29.2 - 35.6) | **-1.6 (-3.0 to -0.2)** | **0.0231** |
| Illinois | 134 | 35.2 (28.9 - 41.5) | 166 | 23.4 (19.8 - 27.0) | **-3.9 (-5.6 to -2.2)** | **<0.0001** |
| Indiana | 26 | 39.9 (24.4-61.6) | 27 | 28.7 (18.2 - 43.1) | -4.9 (-9.6 to +0.1) | 0.0543 |
| Louisiana | 27 | 52.7 (33.4-79.1) | 26 | 37.7 (24.4 - 55.6) | -5.0 (-10.8 to +1.1) | 0.1074 |
| Maryland | 77 | 33.2 (25.8 - 42.1) | 129 | 30.5 (25.2 - 35.8) | -1.3 (-3.2 to +0.7) | 0.1949 |
| Massachusetts | 60 | 29.9 (22.5 - 38.9) | 90 | 24.1 (19.3 - 29.7) | -1.8 (-4.2 to +1.0) | 0.2058 |
| Michigan | 34 | 25.4 (16.9 - 36.6) | 66 | 25.4 (19.5 - 32.6) | -0.9 (-3.6 to +1.8) | 0.5005 |
| Minnesota | 37 | 38.6 (26.4 - 54.5) | 58 | 36.8 (27.5 - 48.1) | **+4.9 (+0.8 to +9.1)** | **0.0181** |
| Missouri | 20 | 35.4 (20.6-56.7) | 23 | 21.1 (13.1 - 32.3) | **-5.2 (-9.7 to -0.5)** | **0.0308** |
| Nevada | 71 | 38.3 (29.2 - 49.4) | 136 | 38.5 (31.8 - 45.1) | +0.2 (-2.0 to +2.4) | 0.8498 |
| New Jersey | 160 | 39.7 (33.0 - 46.5) | 312 | 37.8 (33.5 - 42.0) | -0.7 (-1.9 to +0.5) | 0.2615 |
| New York | 217 | 20.8 (17.9 - 23.6) | 423 | 23.3 (21.1 - 25.6) | -3.7 (-6.0 to -1.3) | 0.8798 |
| North Carolina | 26 | 24.1 (14.7 - 37.3) | 49 | 21.7 (15.7 - 29.1) | -1.3 (-3.9 to +1.3) | 0.3101 |
| Ohio | 33 | 31.2 (20.8 - 45.1) | 57 | 27.7 (20.8 - 36.1) | **-2.6 (-5.1 to -0.1)** | **0.0424** |
| Oklahoma | 21 | 37.3 (22.1-58.9) | 27 | 34.9 (22.1 - 52.4) | +1.0 (-9.2 to +12.4) | 0.8506 |
| Oregon | 25 | 22.8 (14.5 - 34.2) | 55 | 27.6 (20.6 - 36.2) | +2.4 (-0.6 to +5.5) | 0.1176 |
| Pennsylvania | 76 | 41.4 (31.8 - 53.0) | 86 | 23.5 (18.7 - 29.2) | **-3.8 (-6.5 to -1.1)** | **0.0068** |
| Texas | 188 | 36.2 (30.4 - 42.0) | 454 | 39.7 (35.9 - 43.5) | +0.7 (-0.4 to +1.8) | 0.2284 |

| | | | | | | |
|---|---|---|---|---|---|---|
| Utah | 23 | 43.2 (26.4-66.8) | 29 | 32.4 (21.2 - 47.5) | -2.4 (-6.3 to +1.4) | 0.2113 |
| Virginia | 84 | 37.0 (28.7 - 46.9) | 117 | 22.8 (18.6 - 27.1) | **-3.8 (-5.7 to -1.8)** | **0.0002** |
| Washington | 142 | 36.8 (30.5 - 43.1) | 240 | 33.0 (28.8 - 37.3) | +0.9 (-1.3 to +3.2) | 0.4256 |
| Wisconsin | 25 | 43.3 (26.8 - 66.2) | 29 | 27.1 (17.6 - 40.0) | **-4.6 (-8.4 to -0.6)** | **0.0250** |

a AAMR: age-adjusted mortality rates, expressed per 100,000 population

b 95% CI: 95% confidence interval

c APC: Annual Percent Change

d Boldfaced entries represent statistically significant trends

**Table S5. Trends of state-level sepsis-related mortality among Native American individuals, 2010-2019**

| State | 2010 | | 2019 | | | |
|---|---|---|---|---|---|---|
| | **Deaths** | **AAMR[a] (95% CI)[b]** | **Deaths** | **AAMR (95% CI)** | **APC[c],d(95% CI)** | **p value[d]** |
| Alaska | 45 | 64.2 (45.5 - 88.2) | 82 | 91.1 (71.3 - 114.7) | +2.0 (-0.5 to +4.5) | 0.1232 |
| Arizona | 210 | 98.5 (84.2 - 112.7) | 226 | 73.0 (63.1 - 83.0) | **-2.4 (-4.5 to -0.2)** | **0.0376** |
| California | 114 | 27.7 (22.2 - 33.2) | 196 | 35.0 (29.9 - 40.2) | +1.4 (-050 to +3.3) | 0.1389 |
| Michigan | 29 | 54.7 (35.0 - 81.4) | 39 | 47.4 (33.0 - 65.9) | -1.7 (-4.8 to +1.4) | 0.2684 |
| Minnesota | 25 | 68.3 (40.5 - 107.9) | 45 | 76.6 (54.2 - 105.2) | **+5.8 (+1.8 to +10.0)** | **0.0039** |
| Montana | 27 | 61.6 (38.1 - 94.1) | 71 | 130.3 (100.1 - 166.6) | **+9.8 (+6.2 to +13.5)** | **<0.0001** |
| New Mexico | 105 | 73.2 (58.5 - 87.9) | 182 | 91.2 (77.6 - 104.7) | +1.7 (-0.6 to +4.0) | 0.1393 |
| New York | 25 | 19.9 (12.3 - 30.4) | 20 | 11.0 (6.6 - 17.2) | -3.6 (-7.2 to +0.2) | 0.0623 |
| North Carolina | 65 | 74.0 (55.8 - 96.3) | 74 | 51.9 (40.2 - 65.9) | -1.3 (-4.5 to +2.0) | 0.4505 |
| North Dakota | 24 | 126.4 (74.9 - 199.7) | 33 | 112.5 (73.5 - 164.9) | -0.4 (-3.3 to +2.5) | 0.7705 |
| Oklahoma | 206 | 85.6 (73.3 - 98.0) | 311 | 94.8 (83.8 - 105.8) | +1.9 (-0.1 to +3.8) | 0.0503 |
| Oregon | 23 | 45.6 (27.0-72.1) | 21 | 32.6 (19.6 - 50.9) | -2.2 (-6.6 to +2.4) | 0.3451 |
| South Dakota | 46 | 111.5 (78.9 - 153.0) | 78 | 153.7 (119.6 - 194.6) | **+3.4 (+1.0 to +5.8)** | **0.0048** |
| Texas | 22 | 13.3 (7.8-21.3) | 20 | 9.1 (5.4 - 14.4) | -1.0 (-6.6 to +5.0) | 0.7376 |
| Washington | 78 | 90.4 (68.8 - 116.7) | 96 | 79.1 (62.5 - 98.7) | +0.5 (-1.9 to +3.0) | 0.6918 |
| Wisconsin | 28 | 78.7 (48.7 - 120.3) | 43 | 75.9 (54.0 - 103.8) | +1.6 (-2.5 to +5.8) | 0.4546 |

a AAMR: age-adjusted mortality rates, expressed per 100,000 population

b 95% CI: 95% confidence interval

c APC: Annual Percent Change

d Boldfaced entries represent statistically significant trends

**Table S6. Trends of state-level sepsis-related mortality among White individuals, 2010-2019**

| State | 2010 | | 2019 | | | |
|---|---|---|---|---|---|---|
| | Deaths | AAMR[a] (95% CI)[b] | Deaths | AAMR (95% CI) | APC[c,d] (95% CI) | p value[d] |
| Alabama | 2161 | 52.9 (50.7 - 55.2) | 2810 | 59.2 (57.0 - 61.5) | **+1.9 (+1.3 to +2.4)** | **<0.0001** |
| Alaska | 121 | 32.6 (26.3 - 39.0) | 186 | 39.8 (33.8 - 45.7) | **+2.8 (+1.1 to +4.5)** | **0.0014** |
| Arizona | 2331 | 36.9 (35.4 - 38.4) | 2031 | 24.2 (23.2 - 25.3) | **-2.8 (-5.6 to -0.1)** | **0.0489** |
| Arkansas | 1455 | 50.7 (48.0 - 53.3) | 2154 | 64.3 (61.5 - 67.1) | **+2.1 (+0.9 to +3.2)** | **0.0003** |
| California | 13891 | 48.5 (47.6 - 49.3) | 17096 | 50.3 (49.5 - 51.1) | **+0.9 (+0.4 to +1.5)** | **0.0013** |
| Colorado | 1401 | 32.4 (30.6 - 34.1) | 2233 | 38.8 (37.2 - 40.4) | **+3.5 (+2.3 to +4.7)** | **<0.0001** |
| Connecticut | 1922 | 47.8 (45.6 - 50.0) | 2289 | 51.6 (49.4 - 53.8) | **+0.9 (+0.3 to +1.5)** | **0.0018** |
| Delaware | 409 | 48.0 (43.3 - 52.7) | 403 | 37.4 (33.7 - 41.2) | -0.3 (-3.0 to +2.4) | 0.8174 |
| District of Columbia | 64 | 30.9 (23.6 - 39.7) | 61 | 23.2 (17.7 - 30.0) | **-2.5 (-4.8 to -0.1)** | **0.0373** |
| Florida | 7636 | 34.0 (33.2 - 34.8) | 11546 | 40.9 (40.1 - 41.7) | **+2.6 (+2.1 to +3.1)** | **<0.0001** |
| Georgia | 3281 | 52.3 (50.5 - 54.1) | 4413 | 55.0 (53.3 - 56.6) | **+1.0 (+0.6 to +1.4)** | **<0.0001** |
| Hawaii | 139 | 32.4 (26.9 - 37.9) | 116 | 21.5 (17.5 - 25.6) | -1.1 (-3.4 to +1.3) | 0.3718 |
| Idaho | 456 | 29.4 (26.7 - 32.2) | 782 | 38.9 (36.2 - 41.7) | **+2.7 (+1.8 to +3.6)** | **<0.0001** |
| Illinois | 5490 | 47.6 (46.4 - 48.9) | 6238 | 48.1 (46.9 - 49.3) | +0.6 (-0.1 to +1.1) | 0.0547 |
| Indiana | 2954 | 46.0 (44.4 - 47.7) | 4358 | 59.0 (57.2 - 60.8) | **+2.8 (+2.1 to +3.4)** | **<0.0001** |
| Iowa | 1171 | 31.3 (29.5 - 33.1) | 1757 | 43.1 (41.0 - 45.2) | **+4.2 (+3.3 to +5.2)** | **<0.0001** |
| Kansas | 1089 | 36.4 (34.2 - 38.6) | 1725 | 50.5 (48.1 - 53.0) | **+4.4 (+3.2 to +5.6)** | **<0.0001** |
| Kentucky | 2723 | 62.8 (60.4 - 65.2) | 4236 | 83.4 (80.8 - 85.9) | **+4.4 (+3.2 to +5.6)** | **<0.0001** |
| Louisiana | 1857 | 55.6 (60.4 - 65.2) | 2078 | 52.6 (50.3 - 54.9) | +0.6 (-0.4 to +1.5) | 0.2485 |
| Maine | 621 | 36.9 (34.0 - 39.9) | 427 | 22.6 (20.3 - 24.8) | -2.5 (-5.3 to +0.2) | 0.0735 |
| Maryland | 2067 | 47.3 (45.2 - 49.3) | 2214 | 44.6 (42.7 - 46.5) | **-0.9 (-1.5 to -0.2)** | **0.0112** |
| Massachusetts | 3177 | 44.6 (43.0 - 46.2) | 3854 | 47.7 (46.1 - 49.2) | **+1.5 (+0.6 to +2.4)** | **0.0007** |
| Michigan | 4298 | 43.6 (42.3 - 44.9) | 5044 | 44.5 (43.2 - 45.7) | +0.3 (-0.2 to +0.8) | 0.2392 |
| Minnesota | 1479 | 26.6 (25.2 - 27.9) | 2265 | 34.3 (32.9 - 35.8) | **+3.4 (+2.6 to +4.3)** | **<0.0001** |

| | | | | | | |
|---|---|---|---|---|---|---|
| Mississippi | 1181 | 54.2 (51.0 - 57.3) | 1686 | 68.2 (64.9 - 71.5) | **+3.0 (+1.8 to +4.3)** | **<0.0001** |
| Missouri | 2552 | 41.4 (39.8 - 43.0) | 3177 | 44.8 (43.2 - 46.4) | **+0.9 (+0.1 to +1.8)** | **0.0380** |
| Montana | 324 | 28.6 (25.4 - 31.8) | 513 | 37.2 (33.9 - 40.6) | **+4.3 (+2.8 to +5.7)** | **<0.0001** |
| Nebraska | 590 | 29.5 (27.1 - 32.0) | 978 | 43.3 (40.6 - 46.1) | **+5.5 (+4.6 to +6.5)** | **<0.0001** |
| Nevada | 1212 | 56.7 (53.5 - 60.0) | 1493 | 51.6 (48.9 - 54.2) | +0.6 (-0.9 to +2.1) | 0.4222 |
| New Hampshire | 524 | 36.3 (33.2 - 39.5) | 736 | 41.0 (37.9 - 44.0) | **+1.6 (+0.6 to +2.6)** | **0.0019** |
| New Jersey | 5075 | 59.6 (57.9 - 61.2) | 5134 | 55.4 (53.8 - 57.0) | **-0.5 (-0.9 to -0.2)** | **0.0055** |
| New Mexico | 841 | 43.0 (40.1 - 46.0) | 1097 | 47.0 (44.1 - 49.9) | **+1.2 (+0.4 to +1.9)** | **0.0033** |
| New York | 8201 | 45.7 (44.7 - 46.7) | 8546 | 43.1 (42.1 - 44.0) | -0.4 (-1.4 to +0.6) | 0.3887 |
| North Carolina | 4043 | 50.9 (49.3 - 52.5) | 4925 | 48.9 (47.5 - 50.3) | +0.5 (-0.5 to +1.4) | 0.3386 |
| North Dakota | 248 | 30.1 (26.3 - 33.9) | 405 | 45.1 (40.6 - 49.6) | **+4.2 (+2.7 to +5.6)** | **<0.0001** |
| Ohio | 5719 | 47.7 (46.4 - 48.9) | 6477 | 48.2 (47.0 - 49.4) | +0.5 (-0.1 to +1.2) | 0.0986 |
| Oklahoma | 1997 | 56.3 (53.8 - 58.8) | 2846 | 70.1 (67.5 - 72.7) | **+3.5 (+2.6 to +4.4)** | **<0.0001** |
| Oregon | 1310 | 30.9 (29.2 - 32.6) | 1731 | 34.0 (32.4 - 35.7) | **+1.9 (+0.8 to +3.0)** | **0.0007** |
| Pennsylvania | 6840 | 46.0 (44.9 - 47.1) | 7594 | 46.9 (45.8 - 48.0) | **+0.7 (+0.2 to +1.3)** | **0.0110** |
| Rhode Island | 693 | 55.6 (51.4 - 59.9) | 594 | 44.1 (40.5 - 47.8) | **-2.5 (-3.6 to -1.4)** | **<0.0001** |
| South Carolina | 1951 | 51.7 (49.4 - 54.0) | 2864 | 57.2 (55.0 - 59.3) | **+2.1 (+0.9 to +3.3)** | **0.0006** |
| South Dakota | 247 | 25.5 (22.2 - 28.7) | 550 | 50.5 (46.1 - 54.8) | **+7.4 (+5.9 to +9.0)** | **<0.0001** |
| Tennessee | 3212 | 54.3 (52.4 - 56.2) | 4417 | 61.5 (59.6 - 63.3) | **+2.2 (+1.6 to +2.8)** | **<0.0001** |
| Texas | 10959 | 58.2 (57.1 - 59.3) | 14223 | 58.7 (57.7 - 59.7) | **+0.9 (+0.1 to +1.8)** | **0.0305** |
| Utah | 624 | 30.9 (28.4 - 33.3) | 1074 | 40.9 (38.4 - 43.3) | **+3.1 (+1.6 to +4.6)** | **0.0001** |
| Vermont | 251 | 34.0 (29.7 - 38.3) | 269 | 30.5 (26.8 - 34.3) | **-1.8 (-3.3 to -0.3)** | **0.0202** |
| Virginia | 3218 | 50.6 (48.8 - 52.4) | 3431 | 43.6 (42.1 - 45.1) | -0.9 (-2.0 to +0.2) | 0.1196 |
| Washington | 2471 | 39.2 (37.6 - 40.7) | 3533 | 45.3 (43.8 - 46.8) | **+1.8 (+1.1 to +2.5)** | **<0.0001** |
| West Virginia | 1376 | 61.2 (57.9 - 64.4) | 1980 | 81.0 (77.3 - 84.7) | **+3.7 (+3.0 to +4.3)** | **<0.0001** |
| Wisconsin | 1890 | 30.2 (28.9 - 31.6) | 2807 | 39.1 (37.6 - 40.6) | **+3.8 (+2.8 to +4.8)** | **<0.0001** |
| Wyoming | 190 | 33.3 (28.5 - 38.1) | 247 | 36.4 (31.7 - 41.0) | **+4.4 (+1.8 to +7.0)** | **0.0009** |

a AAMR: age-adjusted mortality rates, expressed per 100,000 population

b 95% CI: 95% confidence interval

c APC: Annual Percent Change

d Boldfaced entries represent statistically significant trends

**Table S7. Changes in age-adjusted sepsis-related mortality rates nationally and at across states in 2010 vs 2019**

| Group | Year | AAMR[a] | | | | |
|---|---|---|---|---|---|---|
| | | National mean | State-level median (IQR)[b] | State-level range | Difference in mortality[c] | p value[d] |
| **Black** | | | | | | **0.0032** |
| | 2010 | 79.5 | 77.8 (57.4-89.9) | 31.9-116.8 | 84.9 | |
| | 2019 | 73.0 | 69.6 (57.8-80.5) | 32.7 -104.0 | 71.3 | |
| **Hispanic** | | | | | | 0.1518 |
| | 2010 | 48.9 | 37.8 (27.5-48.3) | 21.9-70.8 | 48.9 | |
| | 2019 | 45.2 | 35.8 (30.4-42.8) | 21.9-65.3 | 43.4 | |
| **Asian** | | | | | | **0.0004** |
| | 2010 | 34.3 | 36.5 (28.0-38.6) | 20.5-52.7 | 32.2 | |
| | 2019 | 30.9 | 27.7 (23.4-33.6) | 19.6-39.7 | 20.1 | |
| **Native American** | | | | | | 0.8564 |
| | 2010 | 51.2 | 70.8 (50.2-88.0) | 13.3-126.4 | 113.1 | |
| | 2019 | 65.5 | 76.3 (41.2-93.0) | 9.1-153.7 | 144.6 | |
| **White** | | | | | | **0.0031** |
| | 2010 | 45.5 | 44.6 (32.5-51.5) | 25.5-62.8 | 37.3 | |
| | 2019 | 48.0 | 45.1 (39.3-52.4) | 21.5-83.4 | 61.9 | |

a AAMR: age-adjusted mortality rate, expressed per 100,000 population

b IQR: interquartile range

c Difference in age-adjusted mortality rate between states with the highest and lowest mortality rate within race and ethnicity groups, as presented in the state-level range column

d p values of comparisons of across-state age-adjusted mortality rate within race and ethnicity groups between 2010 and 2019

**Table S8. Changes in differences of within-state age-adjusted sepsis-related mortality rates between racial and ethnic minority groups and White individuals, nationally and across states in 2010 vs 2019**

| | | Difference in AAMR[a, b, c] between racial and ethnic minorities vs Whites | | | | |
|---|---|---|---|---|---|---|
| **Group** | **Year** | **National mean** | **State-level median (IQR)[d]** | **State-level range** | **Difference in mortality gaps[e]** | **p value[f]** |
| **Blacks** | | | | | | <0.0001 |
| | 2010 | +34.0 | +28.3 (+18.1 to +35.5) | -3.4 to +85.9 | 89.3 | |
| | 2019 | +25.0 | +19.3 (+10.7 to +25.6) | -9.4 to +80.8 | 90.2 | |
| **Hispanics** | | | | | | 0.0003 |
| | 2010 | +3.4 | -0.8 (-12.8 to +5.4) | -28.2 to +14.2 | 70.6 | |
| | 2019 | -2.8 | -7.7 (-17.5 to -4.8) | -32.0 to +6.6 | 38.6 | |
| **Asians** | | | | | | 0.0023 |
| | 2010 | -11.2 | -11.8 (-17.7 to -5.3) | -26.8 to +13.1 | 39.9 | |
| | 2019 | -17.1 | -18.6 (-23.1 to -12.7) | -35.2 to +10.9 | 46.1 | |
| **Native Americans** | | | | | | 0.4037 |
| | 2010 | +5.7 | +30.9 (+12.9 to +49.9) | -44.9 to +96.3 | 141.2 | |
| | 2019 | +17.5 | +35.3 (+0.8 to +50.1) | -49.6 to +96.5 | 146.1 | |

a AAMR: age-adjusted mortality rate, expressed per 100,000 population

b Positive AAMR numbers represent higher age-adjusted sepsis-related mortality rates among a race and ethnic minority group than among White individuals in a given state

c Negative AAMR numbers represent lower age-adjusted sepsis-related mortality rates among a race and ethnic minority group than among White individuals in a given state

d IQR: interquartile range

e Difference in the gap in age-adjusted mortality rate between a racial and ethnic minority group and White individuals, between states with the highest and lowest values, as presented in the state-level range column

f p values of comparisons of differences in within-state age-adjusted mortality rates between each race and ethnic minority group and White individuals between 2010 and 2019

**Table S9. Individual state data of differences in within-state age-adjusted sepsis-related mortality rates - Black vs White individuals, 2010 vs 2019**

| | Difference in AAMR[a, b, c] between Black vs White individuals | |
|---|---|---|
| | Year | |
| State | 2010 | 2019 |
| Alabama | +30.6 | +20.9 |
| Arizona | +18.6 | +8.5 |
| Arkansas | +33.3 | +37.7 |
| California | +28.1 | +22.6 |
| Colorado | +17.5 | +21.9 |
| Connecticut | +16.4 | +10.0 |
| Delaware | +9.1 | +18.5 |
| District of Columbia | +85.9 | +80.8 |
| Florida | +24.3 | +15.9 |
| Georgia | +38.0 | +20.6 |
| Illinois | +51.0 | +28.4 |
| Indiana | +31.8 | +15.0 |
| Iowa | +25.4 | +15.7 |
| Kansas | +22.6 | +28.1 |
| Kentucky | +28.0 | +8.0 |
| Louisiana | +35.7 | +28.3 |
| Maryland | +40.1 | +22.2 |
| Massachusetts | +2.8 | -4.3 |
| Michigan | +55.3 | +40.0 |
| Minnesota | +5.3 | +11.6 |
| Mississippi | +42.0 | +28.8 |
| Missouri | +35.0 | +25.6 |
| Nebraska | +24.0 | +5.9 |

| | | |
|---|---|---|
| Nevada | +16.6 | +11.3 |
| New Jersey | +46.0 | +26.1 |
| New Mexico | +4.7 | -6.7 |
| New York | +2.1 | +5.6 |
| North Carolina | +28.5 | +20.0 |
| Ohio | +35.1 | +14.2 |
| Oklahoma | +33.5 | +10.0 |
| Oregon | +21.7 | +15.2 |
| Pennsylvania | +33.9 | +23.3 |
| Rhode Island | -3.4 | -9.4 |
| South Carolina | +40.8 | +33.5 |
| Tennessee | +29.3 | +25.9 |
| Texas | +31.7 | +21.4 |
| Virginia | +27.4 | +18.3 |
| Washington | +22.6 | +16.6 |
| West Virginia | +6.4 | +9.0 |
| Wisconsin | +38.8 | +23.8 |

a AAMR: age-adjusted mortality rate, expressed per 100,000 population

b Positive AAMR numbers represent higher age-adjusted sepsis-related mortality rates among Black individuals than among Whites in a given state

c Negative AAMR numbers represent lower age-adjusted sepsis-related mortality rates among Black individuals than among White individuals in a given state

**Table S10. Individual state data of differences in within-state age-adjusted sepsis-related mortality rates - Hispanic vs White individuals, 2010 vs 2019**

| State | Difference in AAMR[a, b, c] between Hispanic vs White individuals | |
|---|---|---|
| | Year | |
| | 2010 | 2019 |
| Arizona | +6.0 | +3.1 |
| Arkansas | -11.4 | -32.0 |
| California | +4.0 | +0.4 |
| Colorado | +7.3 | +6.0 |
| Connecticut | +4.4 | -5.7 |
| Florida | -0.8 | -7.2 |
| Georgia | -28.0 | -23.8 |
| Idaho | +5.6 | +3.7 |
| Illinois | +0.3 | -7.9 |
| Indiana | +3.1 | -14.9 |
| Kansas | +1.4 | -7.7 |
| Louisiana | -28.2 | -29.5 |
| Maryland | -25.4 | -16.1 |
| Massachusetts | -11.7 | -11.1 |
| Michigan | +4.9 | -5.2 |
| Minnesota | +5.5 | -1.6 |
| Missouri | -15.5 | -22.9 |
| Nebraska | +13.6 | -4.0 |
| Nevada | -13.0 | -15.7 |
| New Jersey | +7.8 | -5.5 |
| New Mexico | +5.5 | -0.1 |
| New York | -7.6 | -7.5 |

| | | |
|---|---|---|
| North Carolina | -25.8 | -24.4 |
| Ohio | -8.1 | -18.0 |
| Oklahoma | -22.8 | -12.8 |
| Oregon | -3.5 | -7.5 |
| Pennsylvania | +4.0 | -10.1 |
| Rhode Island | -7.2 | -12.4 |
| South Carolina | -26.2 | -29.2 |
| Tennessee | -27.9 | -30.4 |
| Texas | +12.6 | +6.6 |
| Utah | -3.2 | -5.1 |
| Virginia | +14.2 | -20.4 |
| Washington | -12.2 | -4.7 |
| Wisconsin | +2.5 | -6.2 |

a AAMR: age-adjusted mortality rate, expressed per 100,000 population

b Positive AAMR numbers represent higher age-adjusted sepsis-related mortality rates among Hispanic individuals than among White individuals in a given state

c Negative AAMR numbers represent lower age-adjusted sepsis-related mortality rates among Hispanic individuals than among White individuals in a given state

**Table S11. Individual state data of differences in within-state age-adjusted sepsis-related mortality rates - Asian vs White individuals, 2010 vs 2019**

| State | Difference in AAMR[a, b, c] between Asian vs White individuals — Year 2010 | 2019 |
|---|---|---|
| Arizona | -16.4 | -4.6 |
| California | -9.9 | -16.1 |
| Colorado | -6.3 | -16.4 |
| Connecticut | -11.1 | -25.7 |
| Florida | -11.0 | -18.1 |
| Georgia | -17.2 | -22.8 |
| Hawaii | +5.1 | +10.9 |
| Illinois | -12.4 | -24.7 |
| Indiana | -6.1 | -30.3 |
| Louisiana | -2.9 | -14.9 |
| Maryland | -14.1 | -14.1 |
| Massachusetts | -14.7 | -23.6 |
| Michigan | -18.2 | -19.1 |
| Minnesota | +12.0 | +2.5 |
| Missouri | -6.0 | -23.7 |
| Nevada | -18.4 | -13.1 |
| New Jersey | -19.9 | -17.6 |
| New York | -24.9 | -19.8 |
| North Carolina | -26.8 | -27.2 |
| Ohio | -16.5 | -20.5 |
| Oklahoma | -19.0 | -35.2 |
| Oregon | -8.1 | -6.4 |

| | | |
|---|---|---|
| Pennsylvania | -4.6 | -23.4 |
| Texas | -22.0 | -19.0 |
| Utah | +12.3 | -8.5 |
| Virginia | -13.6 | -20.8 |
| Washington | -2.4 | -12.3 |
| Wisconsin | +13.1 | -12.0 |

a AAMR: age-adjusted mortality rate, expressed per 100,000 population

b Positive AAMR numbers represent higher age-adjusted sepsis-related mortality rates among Asian individuals than among White individuals in a given state

c Negative AAMR numbers represent lower age-adjusted sepsis-related mortality rates among Asian individuals than among White individuals in a given state

**Table S12. Individual state data of differences in within-state age-adjusted sepsis-related mortality rates - Native American vs White individuals, 2010 vs 2019**

| State | Difference in AAMR[a, b, c] between Native American vs White individuals | |
|---|---|---|
| | Year | |
| | 2010 | 2019 |
| Alaska | +31.6 | +51.3 |
| Arizona | +61.6 | +48.8 |
| California | -20.8 | -15.3 |
| Michigan | +11.1 | +2.9 |
| Minnesota | +41.7 | +42.3 |
| Montana | +33.0 | +93.1 |
| New Mexico | +30.2 | +44.2 |
| New York | -25.8 | -32.1 |
| North Carolina | +23.1 | +3.0 |
| North Dakota | +96.3 | +67.4 |
| Oklahoma | +29.3 | +24.7 |
| Oregon | +14.7 | -1.4 |
| South Dakota | +86.0 | +96.5 |
| Texas | -44.9 | -46.9 |
| Washington | +51.2 | +33.8 |
| Wisconsin | +48.5 | +36.8 |

a AAMR: age-adjusted mortality rate, expressed per 100,000 population

b Positive AAMR numbers represent higher age-adjusted sepsis-related mortality rates among Native American individuals than among White individuals in a given state

c Negative AAMR numbers represent lower age-adjusted sepsis-related mortality rates among Native American individuals than among White individuals in a given state

**Table S13. Interrupted time series analysis of changes in sepsis-related mortality for the United States population, 2010-2019 (comparison of the 2010-2015 and 2016-2019 periods)**

| Parameter | Coefficients[a] (95% CI)[b] | P value |
|---|---|---|
| Initial time trend (2010-2015) | 2.3 (1.8 to 2.9) | <0.0001 |
| Level (intercept) change | 4.3 (-0.9 to 9.8) | 0.1081 |
| Later time trend (2016-2019) | -2.7 (-7.1 to 2.9) | 0.9820 |
| Change in trends[c] | -4.5 (-6.6 to -2.3) | 0.0001 |

a Coefficients for time trends are expressed as annual percent change; coefficients for level change and change of trends are expressed as percentages; the model data are based on segmented negative binomial regression

b 95% CI: 95% confidence interval

c Change in trends represents change between initial time trend (2010-2015) and later time trend (2016-2019)

**Table S14. Interrupted time series analysis of changes in sepsis-related mortality for the United States population, 2010-2019 (comparison of the 2010-2014 and 2015-2019 periods)**

| Parameter | Coefficients[a] (95% CI)[b] | P value |
|---|---|---|
| Initial time trend (2010-2014) | 2.3 (1.5 to 3.2) | <0.0001 |
| Level (intercept) change | 5.0 (0.1 to 10.2) | 0.0438 |
| Later time trend (2015-2019) | -1.0 (-5.6 to 3.7) | 0.9916 |
| Change in trends[c] | -3.3 (-4.9 to -1.6) | 0.0001 |

a Coefficients for time trends are expressed as annual percent change; coefficients for level change and change of trends are expressed as percentages; the model data are based on segmented negative binomial regression

b 95% CI: 95% confidence interval

c Change in trends represents change between initial time trend (2010-2014) and later time trend (2015-2019)
